# Ionic Liquids in the Aza-Michael Reaction: From Early Imidazolium Salts to Bio-Based Catalytic Media

**DOI:** 10.3390/molecules31040628

**Published:** 2026-02-12

**Authors:** Ignacio M. López-Coca, Shima Ghafouriraz, Carlos J. Durán-Valle, Silvia Izquierdo

**Affiliations:** 1 Department of Organic and Inorganic Chemistry, School of Technology, Universidad de Extremadura, 10003 Cáceres, Spain; 2 Research Institute for Sustainable Land Development (INTERRA), Universidad de Extremadura, 10003 Cáceres, Spain; 3 Department of Organic and Inorganic Chemistry, Faculty of Sciences and IACYS, Universidad de Extremadura, 06006 Badajoz, Spain

**Keywords:** aza-Michael, ionic liquids, task-specific ionic liquids, green and sustainable catalysis, C–N bond formation

## Abstract

The aza-Michael reaction is a fundamental transformation for carbon–nitrogen bond formation, providing efficient access to β-amino carbonyl compounds, nitriles, and related nitrogen-containing building blocks of broad importance in medicinal chemistry and organic synthesis. Over the past two decades, ionic liquids (ILs) have attracted considerable attention as alternative reaction media, promoters, and catalysts for aza-Michael reactions, owing to their distinctive physicochemical properties and tunable structures. This review presents a comprehensive and critical overview of ionic-liquid-mediated aza-Michael reactions, emphasizing the evolution of IL design from early imidazolium-based systems to modern task-specific, supported, and bio-derived ionic liquids. Conventional room-temperature ionic liquids are discussed as non-innocent solvents capable of stabilizing charged intermediates and enhancing electrophilicity, thereby enabling catalyst-free or metal-assisted aza-Michael additions. Subsequent sections focus on task-specific ionic liquids incorporating Brønsted acidic, basic, hydrogen-bond-donating, or bifunctional motifs, highlighting how rational structural design translates into improved activity, selectivity, and substrate scope. Particular attention is devoted to guanidine-, DABCO-, and DBU-based ionic liquids, where mechanistic studies reveal cooperative activation modes rather than simple acid–base catalysis. Recent advances in supported and polymeric ionic liquids are also reviewed, demonstrating effective strategies to combine IL-like reactivity with enhanced recyclability and operational simplicity. Overall, this review clarifies the diverse roles of ionic liquids in aza-Michael chemistry and outlines current challenges and future perspectives toward more sustainable and efficient C–N bond-forming methodologies.

## 1. Introduction

The emergence of Green Chemistry in the early 1990s marked a fundamental shift in the chemical sciences toward the intentional minimization of environmental and human health impacts associated with chemical processes. The U.S. Environmental Protection Agency formally introduced its Pollution Prevention Program in 1991, positioning Green Chemistry as a central strategy within this initiative. Led by Paul Anastas, the program articulated a new vision for chemical design, one in which efficiency, hazard reduction, and sustainability were embedded at the molecular level rather than treated as downstream considerations. The publication of the Twelve Principles of Green Chemistry codified this framework and has since guided research efforts across academic and industrial laboratories [1,2,3,4].

Over the past three decades, this paradigm shift has fostered deep interest in alternative reaction media, energy-efficient methodologies, and catalytic systems that reduce waste and enhance safety. Among these strategies, the exploration of neoteric solvents, most notably ionic liquids (ILs), and, more recently, natural deep eutectic solvents, has become mainstream. These emerging solvent classes have been used extensively to promote a range of organic transformations under milder and more environmentally compatible conditions [5]. Their adoption has nonetheless prompted continuous reassessment of their practical, environmental, and mechanistic attributes; studies frequently reveal both advantages and trade-offs, thereby motivating refinements to improve sustainability profiles and synthetic performance [6]. Such reassessment is particularly relevant for carbon–carbon and carbon–heteroatom bond-forming reactions, which lie at the core of molecular construction and functionalization strategies.

Ionic liquids, broadly defined as salts composed entirely of ions with melting points below 100 °C, have attracted sustained attention due to their distinctive physicochemical properties. These include negligible vapor pressure, thermal and chemical stability, non-flammability, broad liquidus ranges, and high tunability through independent variation of cations and anions [7,8]. Their structural modularity enables design of liquids with tailored polarity, hydrophilicity, Brønsted or Lewis acidity/basicity, hydrogen-bonding capacity, and coordination ability, all of which can be fine-tuned to influence reaction rates, selectivities, solubilities, and catalyst stability. As a result, ILs are used as solvents, reaction promoters, and catalysts in numerous areas of chemistry, in environmental applications, and in pharmaceutical and petrochemical technologies [9,10,11,12,13]. For synthetic organic chemistry, ILs have been particularly effective in reactions involving polar or ionic transition states, including various conjugate additions, cycloadditions, and condensations [14,15,16,17,18]. Many ILs are also commercially available, facilitating their evaluation as replacements for conventional volatile organic solvents [19].

Among the reactions that have benefitted from the application of ionic liquids, the aza-Michael addition occupies a prominent position. The aza-Michael reaction, also known as the 1,4-conjugate addition of nitrogen-centered nucleophiles to electron-deficient alkenes, is a cornerstone method for constructing C–N bonds in contemporary organic synthesis. Typically involving the addition of a primary or secondary amine to activated alkenes such as acrylates or acrylonitrile derivatives, the reaction affords β-amino carbonyl compounds, β-amino esters, β-amino nitriles, and related motifs with high atom economy. These products are privileged intermediates in the synthesis of numerous bioactive molecules, including β-amino acids, β-lactams, amino alcohols, alkaloids, and a diversity of natural product analogs [16,20]. As such, the reaction is widely employed in medicinal chemistry, natural product synthesis, and heterocycle construction.

The aza-Michael reaction is notable for its operational simplicity and wide substrate scope. Both intermolecular and intramolecular variants have been extensively explored, and modern research has produced numerous catalytic strategies that enhance reactivity or enable stereocontrol. Catalytic systems developed over the last several decades include organocatalysts, transition metals, Lewis and Brønsted acids or bases, heterogeneous catalysts, and enzymatic approaches [15,16]. Catalyst-free protocols are also known, particularly when both donor and acceptor are highly activated; in such cases, the reaction can proceed efficiently at ambient temperature under solvent-free conditions, offering one hundred percent atom economy and meeting multiple Green Chemistry criteria. Nonetheless, in many synthetically valuable cases, uncatalyzed reactions are prohibitively slow or require harsh conditions, underscoring the importance of optimized catalytic systems.

Advances in organocatalysis have been especially influential, demonstrating significant improvements in enantioselective transformations via both covalent activation modes (e.g., iminium-ion formation) and noncovalent interactions [21,22,23,24]. Parallel developments have extended the aza-Michael reaction to polymer chemistry [25] and to renewable feedstocks through the use of biobased acceptors [26]. These expansions further highlight the adaptability of the aza-Michael reactions and its continuous relevance to green and sustainable synthesis.

Within this broad context, ionic liquids have emerged as compelling media and catalytic components for aza-Michael chemistry. Since the early 2000s, a growing body of literature has documented IL-mediated or IL-catalyzed aza-Michael reactions, noting improvements in reaction rates, yields, scope, and recyclability compared to benchmark procedures [27,28,29,30,31,32,33,34,35]. The ability of ILs to provide unique microenvironments, through hydrogen-bond donor/acceptor interactions, ion-pairing effects, polarity modulation, acidity/basicity, or coordination, makes them particularly versatile facilitators of conjugate addition chemistry [36,37]. Reported benefits frequently include enhanced nucleophile solubilization, stabilization of polar intermediates, and improved turnover of catalytic species.

At the same time, the role of ILs in organic synthesis must be evaluated through a sustainability lens. Conventional room-temperature ionic liquids (RTILs), such as imidazolium-, pyridinium-, ammonium-, or phosphonium-based salts paired with weakly coordinating anions (BF_4_^−^, PF_6_^−^, NTf_2_^−^, OTf^−^, or halides), represent the second generation of ILs and have been the most widely used in synthetic applications. These salts often display high thermal stability and good solvent properties, but they have been criticized for drawbacks including multi-step synthesis, hygroscopicity, high cost, and concerns about environmental persistence and toxicity. Moreover, some IL cations are known to be chemically non-innocent, potentially engaging in side reactions or generating reactive intermediates upon decomposition [38,39].

The evolution of IL design has therefore proceeded in successive “generations,” each defined by distinct performance targets and structural considerations. The first ionic liquid, ethylammonium nitrate, was described by Walden in 1914, although widespread development began only in the 1980s with electrochemically relevant chloroaluminate systems [40,41,42,43]. From these early materials emerged the second-generation RTILs mentioned above, many of which have been thoroughly explored as solvents or catalysts for the aza-Michael reaction. The third generation comprises task-specific ionic liquids (TSILs) engineered to incorporate functional groups that directly influence catalysis, such as incorporated guanidinium units, sulfonic acid moieties, superbasic species, metal-containing anions, or polymer-supported ionic liquid phases. These TSILs offer enhanced control over Brønsted or Lewis acidity, hydrogen-bonding capacity, or nucleophile activation [6].

More recently, a fourth generation of bio-sourced and biocompatible ionic liquids has emerged, aligned with the broader ambitions of Green Chemistry. These include cholinium-based ILs, amino acid-derived ILs, protic ILs generated from simple biogenic ions, and other materials designed to exhibit low toxicity, biodegradability, and minimal environmental impact [6]. Their potential advantages for aza-Michael chemistry, and for catalysis more broadly, are increasingly recognized, especially given their compatibility with renewable feedstocks and environmentally sensitive applications.

While numerous reviews have addressed aspects of aza-Michael chemistry, including mechanistic foundations, organocatalytic strategies, and asymmetric variants, ionic liquids are often treated only tangentially within these broader discussions. Conversely, existing reviews on ionic liquids typically emphasize general synthetic applications, electrochemistry, separations, or materials science, without providing a systematic comparative analysis of ILs specifically in aza-Michael reactions. Although reviews on magnetic ionic liquids and sonochemical synthesis are available, they offer only limited insight into the aza-Michael reaction, particularly regarding the role of ionic liquids, which is discussed only incidentally [44,45]. As a result, a focused assessment of the role of ILs, across structural generations and functional categories, in promoting aza-Michael reactions remains lacking.

This review addresses that gap by examining IL-mediated aza-Michael chemistry through a generational classification, highlighting the distinct capabilities and limitations associated with each IL type. We survey experimental studies and key reviews to provide a cohesive analysis of the roles that ILs play as solvents, reagents, and catalysts. Throughout, we evaluate these systems from the perspective of green chemistry metrics, including atom economy, energy efficiency, intrinsic hazard, waste reduction, and potential environmental impacts. By consolidating insights across two decades of research, this review aims to clarify the contributions of ionic liquids to aza-Michael chemistry and to identify opportunities for future innovations in sustainable C-N bond formation.

## 2. Conventional Ionic Liquids

### 2.1. Neutral Imidazolium Ionic Liquids

**Scheme 1:**
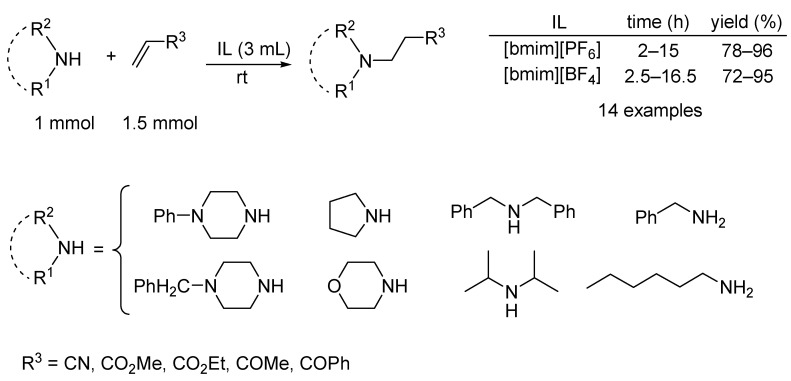
Aza-Michael reaction in [bmim][PF_6_] and [bmim][BF_4_].

The first account on the use of ILs as reaction medium for the aza-Michael reaction was published by Yadav and colleagues in 2003 [27]. In their seminal paper, they demonstrated the application of ionic liquids as novel, environmentally benign reaction media for aza-Michael reactions, achieving facile synthesis of β-amino compounds under mild, catalyst-free conditions. The study employed 1-butyl-3-methylimidazolium hexafluorophosphate ([bmim][PF_6_]) and 1-butyl-3-methylimidazolium tetrafluoroborate ([bmim][BF_4_]) as both solvent and promoter, with [bmim][PF_6_] demonstrating superior performance in terms of conversion rates and reaction times (Scheme 1).

The methodology exhibited broad substrate scope, successfully accommodating various electron-deficient α,β-unsaturated compounds (ethyl acrylate, methyl acrylate, acrylonitrile, methyl cinnamate, and cyclohexenone) and diverse amines (primary, secondary, aliphatic, aromatic, and sterically hindered amines). The reactions proceeded smoothly at room temperature without acid or base catalysts, yielding corresponding β-amino compounds in excellent yields (78–96%) with high 1,4-selectivity. Notably, secondary amines generally provided higher yields than primary amines, while sterically hindered amines also delivered excellent results.

Comparative studies revealed significant advantages over conventional solvents. For instance, the conjugate addition of benzylamine to methyl acrylate in [bmim][PF_6_] afforded the Michael adduct in 92% yield within 8.0 h, compared to 75% yield (with 15% bis-adduct formation) after 24 h in methanol, and 70% yield (7:3 mono-:bis-adduct ratio) in water. The enhanced reactivity in ionic liquids was attributed to their high polarity, which activated the enones and significantly reduced reaction times while improving yields and selectivity. Yet, there is some controversy because there may be some acid catalysis arising from the hydrolysis of the PF_6_^−^ anion due to its water content. The tetrafluoroborate anion presents no such issue, since it is common knowledge that it is more water stable than the PF_6_^−^. This paper does not report the water content of the ionic liquid; therefore, it remains uncertain whether protons generated from hexafluorophosphate hydrolysis are the actual catalytic species. The protocol offered additional practical advantages including simple product isolation through diethyl ether extraction and excellent recyclability of the ionic liquid medium. The recovered [bmim][PF_6_] maintained similar catalytic activity and product purity through multiple reaction cycles, demonstrating the sustainability of this approach. The anion nature strongly influenced the ionic liquid efficiency, with PF_6_^−^ outperforming BF_4_^−^ in most cases.

This pioneering work established ionic liquids as promising green alternatives to volatile organic solvents for aza-Michael reactions, from the perspective of their negligible volatility, providing access to valuable β-amino intermediates crucial for β-amino acid and β-lactam antibiotic synthesis while minimizing environmental impact through the elimination of toxic catalysts and volatile solvents.

**Scheme 2:**
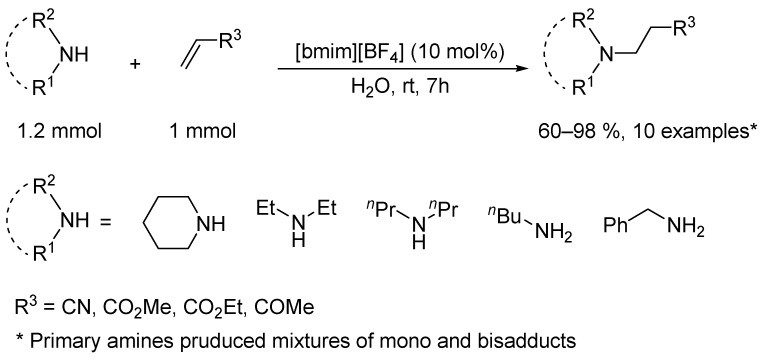
Aza-Michael reaction catalyzed by [bmim][BF_4_] in water.

Soon after, Xu and co-workers demonstrated that simple quaternary ammonium salts and the hydrophilic ionic liquid [bmim][BF_4_] can catalyze the addition of aliphatic amines to α,β-unsaturated compounds in water under remarkably mild conditions (room temperature, atmospheric pressure) [28]. The method afforded β-amino carbonyl products in high to near-quantitative yields and, importantly, the ionic liquid could be recovered and was reused five times without loss of activity, and the product was obtained with similar yields, underscoring its potential for sustainable synthesis (Scheme 2).

When primary benzyl and *n*-butyl amines were employed as starting materials, mixtures of mono- and bis-adducts were obtained in all cases, with reported isolated yield ratios ranging from 94:2 to 60:36 (mono/bis). However, under an amine-to-alkene stoichiometry of 1.2:1.0, an isolated yield of 60:36 (mono/bis) is not feasible. The study was notable for its green chemistry aspects: water as the reaction medium, avoidance of transition metals, and the recovery/reuse of the ionic liquid. At the same time, the scope revealed inherent limitations. The catalytic system proved effective only with aliphatic amines, while aromatic amines failed to undergo addition, highlighting selectivity that could be useful synthetically but also pointing to constraints in substrate generality. Compared with prior metal-based catalysts, the approach is simpler and cheaper, but it lacks the broad applicability and fine-tuned reactivity seen in later developments.

These reports helped establish ionic liquids as viable catalysts and media for aza-Michael chemistry, laying groundwork for subsequent efforts toward asymmetric variants and broader substrate classes [27,28].

Due to its ease of production, the catalytic activity found and its stability against hydrolysis, [bmim][BF_4_] has been further studied under other conditions in intramolecular aza-Michael reactions, activated by microwaves [46] or not [32], or in intramolecular addition to steroidal Michael acceptors [47].

**Scheme 3:**
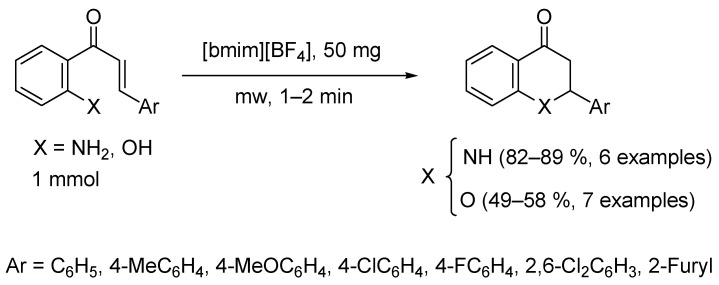
Intramolecular mw-promoted hetero-Michael addition in [bmim][BF_4_].

Kumar and co-workers reported one of the earliest systematic demonstrations of the efficiency of imidazolium ionic liquids in promoting intramolecular hetero-Michael cyclizations (aza-, and oxa-Michael). In their study, 2′-aminochalcones and 2′-hydroxychalcones underwent smooth cyclization to the corresponding 2-aryl-2,3-dihydroquinolin-4(1*H*)-ones and chromen-4-ones, respectively, when treated with 1-butyl-3-methylimidazolium tetrafluoroborate ([bmim][BF_4_]) under microwave irradiation [46]. The combination of microwave heating and the excellent dielectric properties of the ionic liquid led to reaction times on the order of 1–2 min, with moderate to high isolated yields across a range of substituted substrates (Scheme 3).

Mechanistic considerations, reinforced by computational analysis, indicated that the ionic liquid environment stabilizes the polar transition states and enhances the nucleophilicity of the internal amino or hydroxy group, thereby facilitating rapid cyclization. In fact, when polar organic solvents such as DMF and DMSO were tested under microwave irradiation, poor yields were obtained. Moreover, in absence of ionic liquid, the reaction did not proceed under microwave irradiation. Notably, the system allowed for the recovery and reuse of [bmim][BF_4_] without loss of catalytic efficiency, underscoring its operational practicality. This study established a valuable precedent for IL-assisted, microwave-promoted aza-Michael processes and highlighted the synergistic interaction between microwave energy and ionic media.

**Scheme 4:**
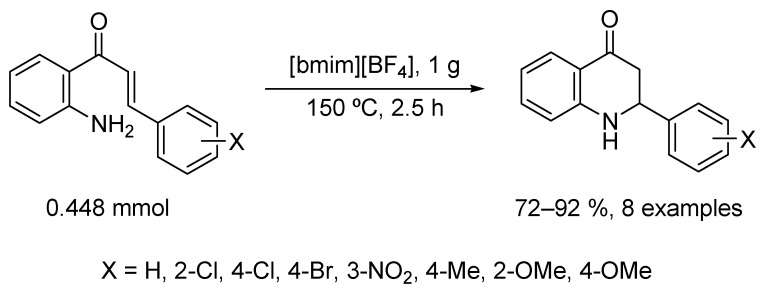
Intramolecular thermal aza-Michael in [bmim][BF_4_].

Chelghoum and collaborators expanded the scope of ionic-liquid-mediated aza-Michael chemistry by showing that 2′-aminochalcones can undergo efficient intramolecular cyclization in [bmim][BF_4_] without the need for any additional catalyst [32]. Conducted at 150 °C, the thermal isomerization proceeds cleanly to yield 2,3-dihydroquinolin-4(1*H*)-ones in good to excellent yields (70–92%), with broad tolerance toward both electron-donating and electron-withdrawing aromatic substituents. (Scheme 4) Other ILs ([bpy][BF_4_], and [bmim][PF_6_] were tested as well, with slightly poorer outcome [32].

A salient feature of this work is the use of the ionic liquid not merely as a solvent but also as a reaction promoter, presumably through stabilization of polar intermediates in the Michael addition pathway. The ionic liquid was shown to be recoverable and reusable for three cycles, with only a gradual decline in performance. Structural confirmation through X-ray crystallography further substantiated the reliability of the method. The study therefore demonstrates that ionic liquids alone can serve as recyclable, mild, and efficient media for intramolecular aza-Michael additions, obviating the need for conventional acidic or basic catalysts.

**Scheme 5:**
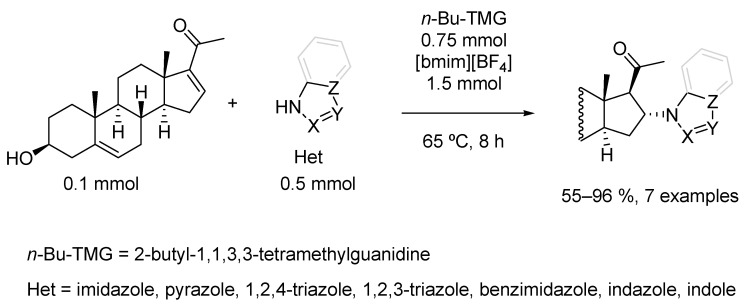
Aza-Michael addition of 16-dehydropregnenolone in [bmim][BF_4_].

In a significant conceptual advance, Maksó et al. introduced a fully recyclable, CO_2_-switchable ionic-liquid-based platform for the intermolecular aza-Michael addition of diverse *N*-heterocycles to the steroidal Michael acceptor 16-dehydropregnenolone (16-DHP) [47]. Their protocol employs [bmim][BF_4_] as the solvent and 2-butyl-1,1,3,3-tetramethylguanidine (*^n^*Bu-TMG) as the catalyst, taking advantage of the ability of guanidine bases and *N*-heterocycles to form reversible ionic species in the presence of CO_2_ (Scheme 5).

This strategy enables the simultaneous recycling of the solvent, the catalyst, and the excess nucleophilic reagent, representing a rare example of triple-component recycling in aza-Michael chemistry. Introduction of CO_2_ post-reaction converts the base and unreacted heterocycle into ionic forms that remain selectively in the IL phase, thereby allowing clean extraction of the neutral steroidal products with apolar solvents. Subsequent removal of CO_2_ regenerates the active catalytic system. The methodology demonstrates high conversions across a wide range of *N*-heterocycles, including imidazole, pyrazole, triazoles, and benzimidazole, while more weakly nucleophilic reagents (e.g., indole, indazole) require adjustment of basicity to maintain recyclability.

Beyond synthetic efficiency, this work provides a model for sustainable aza-Michael methodology, showcasing how IL media can be integrated with reversible ionic systems to minimize waste and avoid sacrificial reagents.

**Scheme 6:**
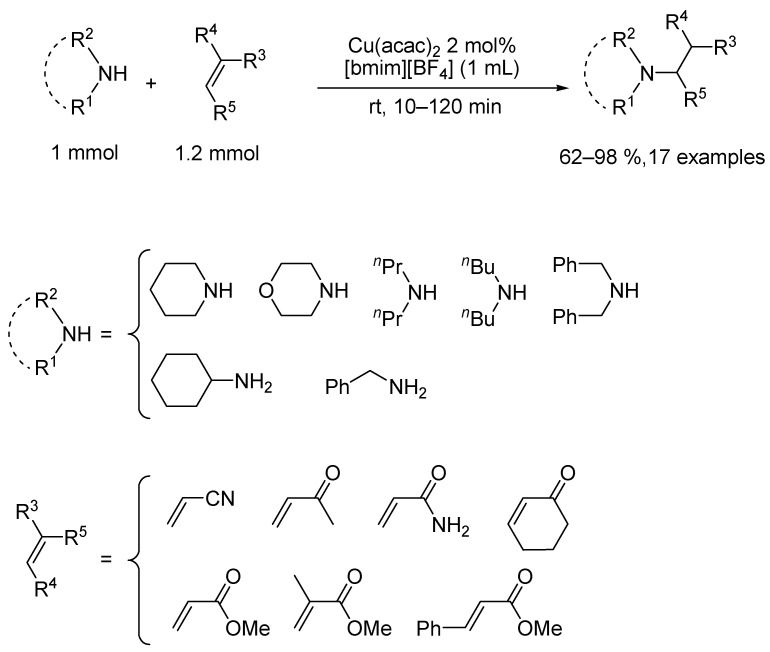
Aza-Michael reaction in Cu(acac)_2_ Immobilized in [bmim][BF_4_].

Taking the use of conventional ILs one step-further, quite early, Kantam and co-workers reported a seminal example of combining a homogeneous Lewis acid with an ionic liquid to create a highly efficient and recyclable catalytic system for aza-Michael reactions [48]. In this study, copper(II) acetylacetonate was immobilized in imidazolium-based ionic liquids ([bmim][BF_4_] and [bmim][PF_6_]) and applied to the conjugate addition of a wide range of aliphatic amines to α,β-unsaturated carbonyl compounds and nitriles under mild, room-temperature conditions. The protocol afforded the corresponding β-amino carbonyl compounds and β-aminonitriles in excellent yields, typically within minutes, and consistently outperformed analogous reactions conducted in conventional solvents or in ionic liquids without a metal catalyst (Scheme 6).

Notably, the catalytic system exhibited broad substrate scope, accommodating esters, enones, acrylonitrile, and amides, with secondary amines generally giving higher yields than primary amines. The ionic liquid phase containing Cu(acac)_2_ could be readily separated from the products by simple extraction and reused for multiple cycles without significant loss of activity, and the methodology was demonstrated to be scalable. This work represents an early and influential contribution to sustainable catalysis, illustrating how ionic liquids can serve simultaneously as solvent and immobilization medium to enable efficient, recyclable metal-catalyzed aza-Michael reactions.

**Scheme 7:**
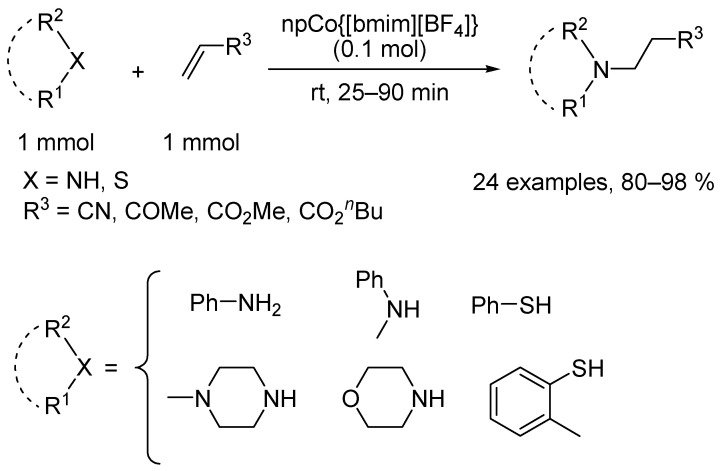
Aza- and thia-Michael reaction catalyzed by [bmim][BF_4_] stabilized cobalt nanoparticles.

A further advancement in the use of [bmim][BF_4_] is reported by Dewan et al. describing an efficient and green catalytic system based on ionic liquid-stabilized magnetic cobalt nanoparticles for aza- and thia-Michael addition reactions [49]. In this work, cobalt nanoparticles stabilized by a thin electrosteric layer of the ionic liquid [bmim][BF_4_] were shown to catalyze the conjugate addition of amines and thiols to a variety of activated α,β-unsaturated electrophiles, including acrylonitrile, methyl vinyl ketone, and acrylate esters, under solvent-free and ambient conditions (Scheme 7). The reactions proceeded rapidly at room temperature, typically within minutes, affording the corresponding β-amino and β-thio carbonyl or nitrile adducts in high to excellent isolated yields, while clearly outperforming bare, non-stabilized cobalt nanoparticles, which suffered from low activity and poor recyclability.

A key feature of this methodology is the magnetic separability and remarkable reusability of the catalyst. The ionic liquid coating not only prevented nanoparticle aggregation and degradation but also preserved catalytic activity over multiple cycles, with up to seven to eight consecutive runs showing negligible loss in efficiency. The study further demonstrated that catalytic performance depended on both particle size and catalyst loading, with optimal activity observed for nanoparticles around 30 nm at 5 mol% loading. Overall, this contribution highlights the synergistic combination of ionic liquids and magnetic nanocatalysts as a robust, recyclable, and environmentally benign platform for Michael-type reactions, aligning well with the principles of green and sustainable chemistry.

These studies collectively demonstrate that 1-butyl-3-methylimidazolium tetrafluoroborate ([bmim][BF_4_]) is a remarkably versatile ionic liquid whose physicochemical properties confer distinct catalytic or promotional effects depending on the reaction system, activation mode, and substrate class. In early contributions, [bmim][BF_4_] was shown to exert pronounced solvent effects that enhance the electrophilicity of α,β-unsaturated carbonyl compounds. In these cases, the ionic liquid functions as more than an inert reaction medium; it polarizes the conjugated π-system, stabilizes key transition states, and significantly accelerates aza-Michael reactions that would otherwise require harsh conditions or the presence of Lewis acid catalysts [27,28]. Subsequent intramolecular studies further highlighted the capacity of [bmim][BF_4_] to promote ring-closing aza-Michael reactions leading to nitrogen-containing heterocycles, either under microwave irradiation or via conventional thermal activation, without the need for additional catalysts [32,46]. More recently, a highly advanced and multifunctional application of [bmim][BF_4_] was reported [47], employing it not only as an effective reaction medium for the challenging intermolecular aza-Michael addition of diverse *N*-heterocycles to 16-dehydropregnenolone, but also as a central component of a CO_2_-switchable and fully recyclable catalytic system. In combination with the strong guanidine base *^n^*Bu-TMG, the ionic liquid enables high conversions even with weakly nucleophilic heterocycles by stabilizing ionic intermediates and enhancing the electrophilicity of the steroidal Michael acceptor. Notably, post-reaction introduction of CO_2_ converts both the catalyst and excess nucleophile into ionic species that remain confined within the [bmim][BF_4_] phase, allowing selective extraction of the neutral steroidal products using apolar solvents. Subsequent CO_2_ removal regenerates the active catalyst–solvent–reagent system, enabling multiple reaction cycles with only the addition of fresh substrate. This work represents a state-of-the-art application of ionic-liquid technology, illustrating how [bmim][BF_4_] can be integrated into sophisticated circular methodologies that combine high efficiency, chemoselectivity, and minimal waste generation, thereby significantly advancing the sustainability profile of aza-Michael chemistry.

### 2.2. Basic Conventional ILs

**Scheme 8:**
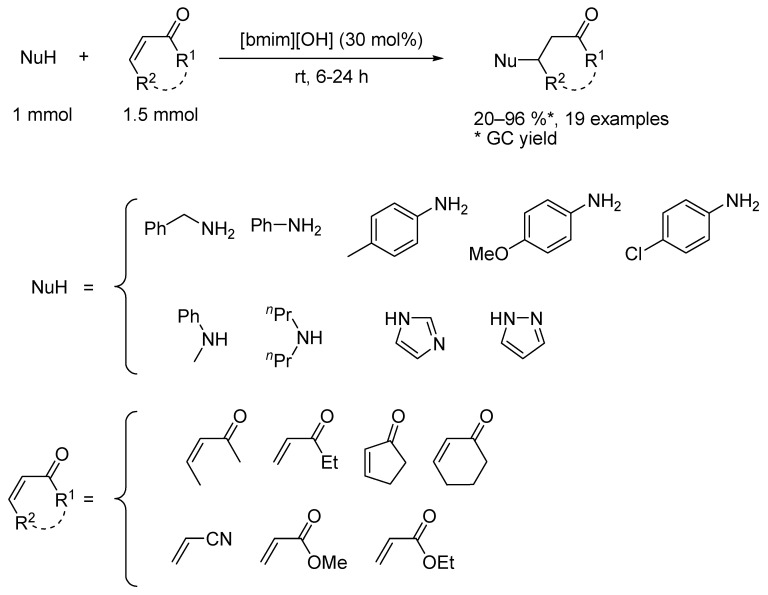
Aza-Michael reaction catalyzed by [bmim][OH].

One of the earliest studies of IL-mediated aza-Michael additions employed the basic imidazolium hydroxide ([bmim]OH) as a catalyst for the addition of aromatic amines and *N*-heterocycles to cyclic or acyclic ketones under neat conditions. The catalyst could be easily recovered after completion of the reaction and reused three times [50] (Scheme 8).

**Scheme 9:**
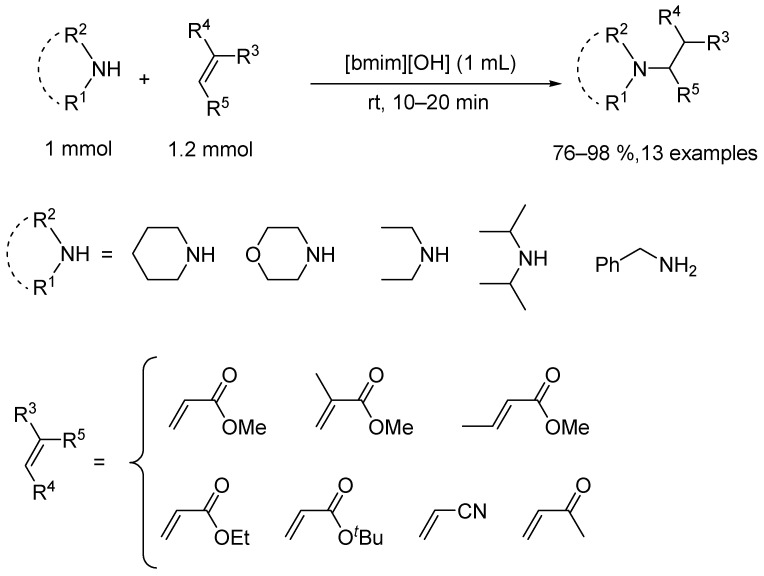
Aza-Michael reaction in neat [bmim][OH].

Xu and co-workers showed that [bmim]OH can act simultaneously as reaction medium and base catalyst for the addition of primary and secondary amines to α,β-unsaturated acceptors [51]. The reaction proceeds to completion in just 10–20 min under neat [bmim][OH] with excellent isolated yields; in contrast, the same reaction in THF or DMSO without IL afforded ≤ 30% product after 48 h (Scheme 9).

The aza-Michael addition between piperidine and methyl acrylate was performed on a larger scale (200 mmol) using [bmim][OH] (5 mL) as the reaction medium. Complete conversion was achieved within 15 min, as confirmed by TLC. The excess methyl acrylate was removed by evaporation and subsequently recovered for reuse. The desired 1,4-adduct was then isolated by direct distillation from the reaction vessel under reduced pressure, affording the product in 99% yield. Importantly, the remaining ionic liquid was reused directly without any further treatment. The reaction was repeated for eight consecutive cycles with no loss of consistent catalytic activity.

These studies established several recurring features of second-generation ILs in aza-Michael chemistry, such as their dual role as both basic catalyst and high-boiling solvent, high efficiency with short reaction times and high yields under mild conditions, and reusability of the IL phase. Its drawbacks can be the need for relatively high IL loadings (often ≥30 mol%), cost, and potential issues of toxicity.

**Scheme 10:**
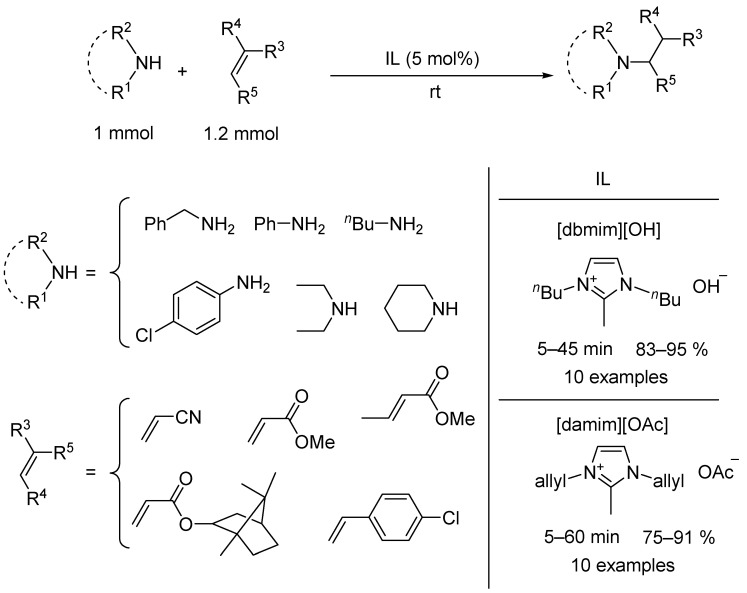
Aza-Michael reaction in neat water-stable basic ILs.

Later, Boruah and Borah reported water-stable 1,3-dialkyl-2-methylimidazolium hydroxide and acetate as basic catalysts for aza-Michael additions in neat conditions, emphasizing high activity and easy recycle. In some aspects, these ILs can be on the blurred line that divides conventional or second-generation ILs and task-specific or third generation ILs [52]. This work emphasizes materials-style characterization (FT-IR, NMR, elemental analysis) and links physicochemical properties to catalytic performance in aza-Michael additions under solvent-free conditions. For the catalytic evaluation, five ionic liquids exhibiting higher thermal stability and lower hygroscopicity were selected to assess their efficiency as catalysts with no solvent but the IL. Excellent product yields were achieved with within 5–7 min (Scheme 10).

## 3. Task-Specific Ionic Liquids

### 3.1. Acidic TSILs

Among various classes of ionic liquids, acidic ionic liquids (AILs) and Brønsted acidic task-specific ionic liquids (TSILs) have attracted particular attention. These systems combine the advantages of homogeneous mineral acids and heterogeneous solid acids while avoiding issues related to corrosion, volatility, and waste generation. The incorporation of acidic functional groups, such as –SO_3_H, into the ionic liquid structure enables efficient activation of electrophilic substrates and stabilization of reaction intermediates. However, many reported TSILs are based on imidazolium cations and halogen-containing anions, which are relatively expensive and raise environmental concerns [30,53]. Therefore, the design of inexpensive, halogen-free, and recyclable acidic ionic liquids represents an important step toward greener and more sustainable aza-Michael protocols. Recent studies have demonstrated that such ionic liquids can effectively promote aza-Michael additions under mild conditions, offering high yields, broad substrate scope, and excellent recyclability [53,54,55,56]. These advances highlight the growing potential of acidic ionic liquids as multifunctional media that act simultaneously as solvent and catalyst, aligning well with the principles of green chemistry. In this context, the following section summarizes representative examples of acidic ionic liquids that have been successfully employed in aza-Michael reactions. These examples highlight the influence of ionic liquid structure on catalytic efficiency, reaction scope, and mechanistic behavior, providing a clear framework for understanding their role in promoting efficient and sustainable aza-Michael transformations.

**Scheme 11:**
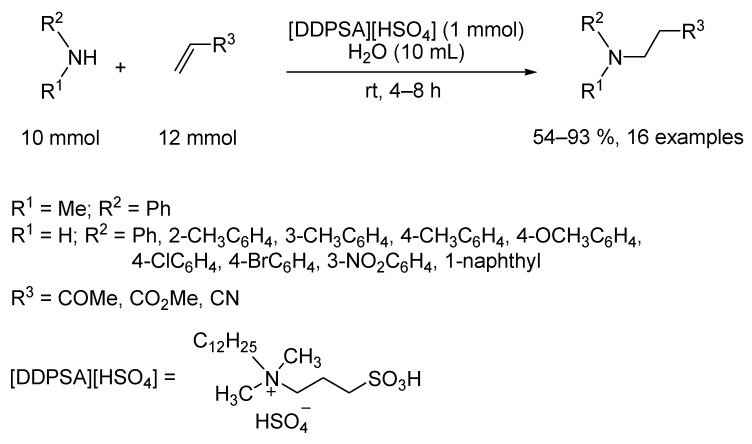
Aza-Michael reaction catalyzed by acidic [DDPSA][HSO_4_].

A SO_3_H-functional, halogen-free Brønsted acidic ionic liquid, [DDPSA][HSO_4_], was introduced by Liu et al. as an efficient and reusable catalyst for the aza-Michael reaction [30]. In this work, they demonstrate that this ionic liquid can effectively catalyze the aza-Michael addition of aromatic amines to α,β-unsaturated compounds under mild conditions (Scheme 11).

To investigate the mechanistic behavior of the system, the addition of aniline to acrylonitrile was selected as the representative transformation. Screening of several solvents demonstrated that the reaction proceeds more efficiently in polar media, consistent with the stabilization of the developing charge during nucleophilic attack. Although polar organic solvents performed well, conducting the reaction in water was also examined due to its environmental and economic advantages.

The role of the acidic ionic liquid [DDPSA][HSO_4_] was then evaluated. In the absence of the catalyst, only traces of product were observed, indicating that activation of the Michael acceptor is essential. Increasing the catalyst loading resulted in a clear improvement in conversion, with 10 mol% providing the optimal balance between activity and efficiency. A key mechanistic feature of this system is the dual function of the ionic liquid: protonation enhances the electrophilicity of the α,β-unsaturated substrate, while the organized ionic environment facilitates nucleophilic attack by the amine. This combination enables the reaction to proceed smoothly under mild conditions. The recyclability of the catalyst further supports its mechanistic robustness. After product extraction, the aqueous catalytic phase could be reused multiple times without noticeable loss of activity, demonstrating that the catalytic species remains intact throughout the reaction cycle. With optimized conditions established, the method was extended to various aromatic amines. Substrates bearing electron-donating or electron-withdrawing substituents participated effectively, and even sterically hindered or secondary amines reacted efficiently. This broad functional-group tolerance highlights the stability and adaptability of the catalytic system.

**Scheme 12:**
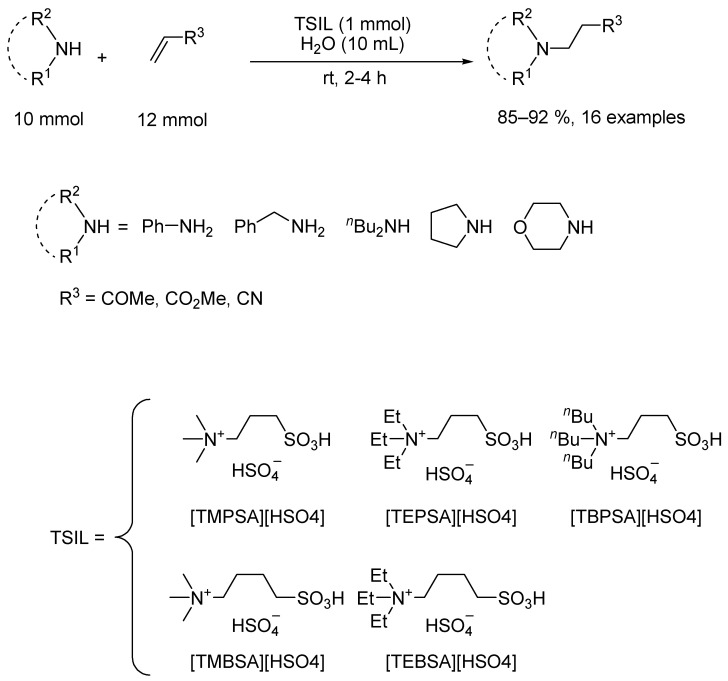
Acidic TSILs as catalysts for the aza-Michael addition.

Liu and co-workers reported an efficient and environmentally benign aza-Michael protocol employing SO_3_H-functionalized, halogen-free Brønsted acidic ionic liquids as recyclable catalysts in water [53]. In this study, a series of task-specific ionic liquids (TSILs) were designed to combine the advantages of solid acids and mineral acids while avoiding halogenated anions and expensive imidazolium-based cations (Scheme 12).

These TSILs effectively catalyze the conjugate addition of amines to α,β-unsaturated carbonyl compounds and nitriles, affording β-amino derivatives in high yields under mild, room-temperature conditions.

Catalyst screening revealed that all investigated SO_3_H-functionalized ionic liquids were active, with [TMPSA][HSO_4_] identified as the most efficient and economically favorable catalyst. The reactions proceed smoothly in water without the need for organic co-solvents, highlighting the strong Brønsted acidity and good water compatibility of the ionic liquid. In the absence of the TSIL catalyst, no conversion was observed, confirming its essential role in activating the Michael acceptor toward nucleophilic amine addition. The substrate scope is broad and includes aromatic amines (e.g., aniline), benzylamines, and secondary aliphatic amines, which react efficiently with methyl acrylate, acrylonitrile, and methyl vinyl ketone. Both primary and secondary amines afforded the corresponding β-amino carbonyl or nitrile products in good to excellent isolated yields, typically within 2–4 h at room temperature. Notably, the method tolerates structural variation in both the nucleophile and the Michael acceptor, underscoring its synthetic versatility. Product isolation is achieved by simple extraction with ethyl acetate, while the aqueous phase containing the ionic liquid catalyst can be reused directly. The catalyst was recycled at least five times without any appreciable loss of activity, demonstrating excellent operational stability and recyclability.

**Scheme 13:**
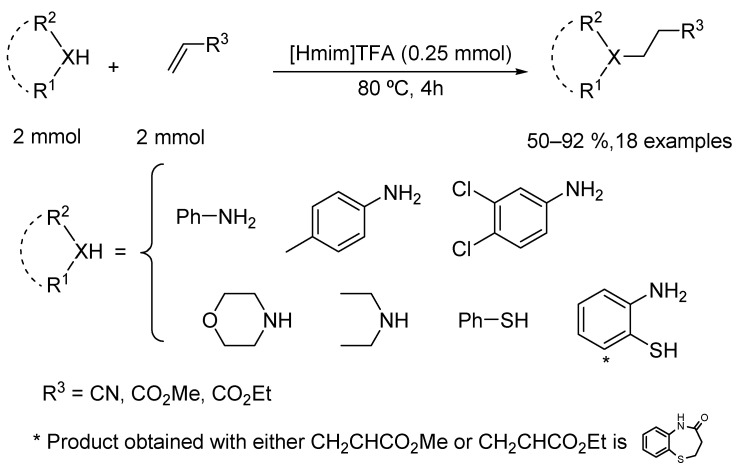
Aza-Michael in 1-methylimidazolium trifluoroacetate ([Hmim][TFA]).

Dabiri and co-workers reported an ecofriendly and efficient protocol for hetero-Michael addition reactions using the acidic ionic liquid 1-methylimidazolium trifluoroacetate ([Hmim][TFA]) as both catalyst and reaction medium [54]. The system enables the conjugate addition of nitrogen- and sulfur-based nucleophiles to a wide range of α,β-unsaturated carbonyl compounds and nitriles under solvent-free conditions. Reactions are typically performed at 80 °C and afford the corresponding Michael adducts in good to excellent yields within relatively short reaction times (Scheme 13).

A notable feature of this methodology is its effectiveness with aromatic amines. Both anilines and heterocyclic amines, as well as thiophenols and bifunctional nucleophiles, were successfully employed. In particular, the use of 2-aminothiophenol led to tandem addition–cyclization processes, affording seven-membered *N*,*S*-heterocycles (benzothiazepinone derivatives) in high yields, highlighting the synthetic versatility of the ionic-liquid-mediated system. From a green chemistry perspective, [Hmim][TFA] plays a dual role by activating the Michael acceptor through Brønsted acidity while simultaneously acting as a non-volatile, reusable reaction medium. Product isolation is straightforward and achieved by extraction with diethyl ether, after which the ionic liquid can be recovered and reused at least three times without significant loss of catalytic activity. This work demonstrates the potential of acidic imidazolium-based ionic liquids as sustainable media and catalysts for aza-Michael and related hetero-Michael reactions. The broad substrate scope, compatibility with aromatic amines, mild conditions, and catalyst recyclability make this approach particularly relevant for inclusion in reviews focused on ionic liquids in green conjugate addition chemistry.

**Scheme 14:**
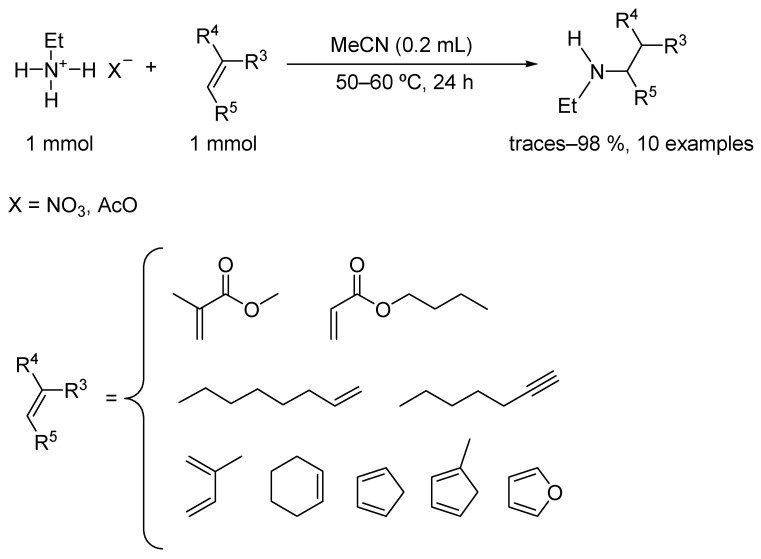
Addition reaction of PILs to unsaturated substrates.

Bravo, et al. presented a study on the role of alkylammonium-based protic ILs (PILs) containing nitrate or acetate counterions as reagents in nucleophilic addition reactions. All PILs were prepared by neutralization of amine with suitable acids [55]. This study reveals that, with the exception of tributylammonium nitrate, most of these ILs contain significant and tunable amounts of free acid and free amine, which directly influence their catalytic behavior. In the context of aza-Michael chemistry, PILs were applied to the nucleophilic addition of amines to α,β-unsaturated esters to form β-amino esters. Acetate-based PILs, particularly ethylammonium acetate, proved especially effective for these reactions, affording β-amino esters in good yields under mild conditions (50–60 °C, 24 h). Importantly, the moderate acidity of the acetate counterion was sufficient to catalyze the aza-Michael addition while suppressing undesired polymerization of the acrylate substrates, a common issue under stronger acidic conditions. In contrast, if the target molecule is the allylic amine obtained by reaction with conjugated dienes, PILs derived from primary and secondary amines, with nitrate as counterion, are more suitable (Scheme 14).

**Scheme 15:**
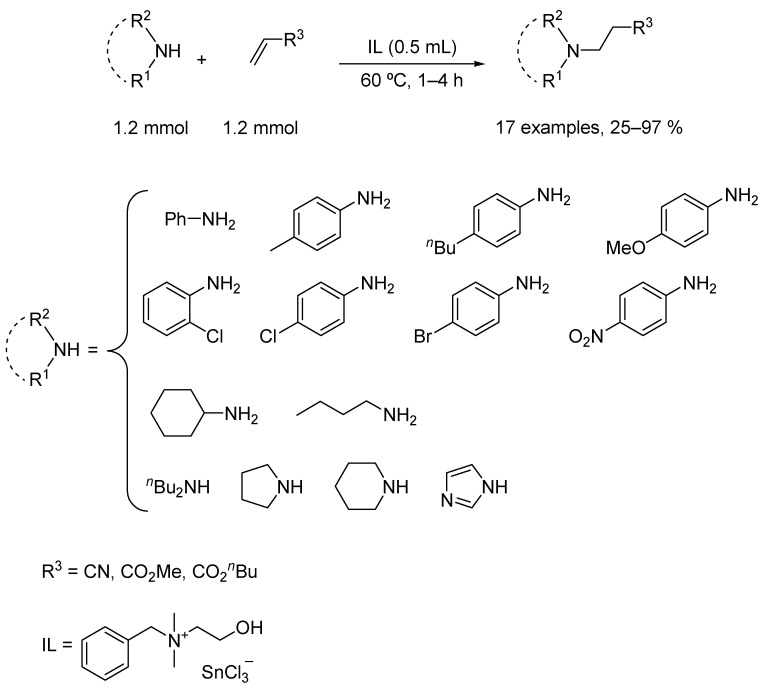
Aza-Michael reaction in acidic tin-based IL.

Recently, Tajik and co-workers reported the development of a novel Lewis acidic ionic liquid (AIL) and its application as an efficient catalyst and reaction medium for aza-Michael addition reactions [56]. The acidic ionic liquid was synthesized via a straightforward, one-pot procedure involving benzyl chloride, 2-(dimethylamino)ethanol, and SnCl_2_, affording a tin-containing ionic liquid with combined Brønsted and Lewis acidic properties. Comprehensive characterization by FT-IR spectroscopy, SEM, EDS, and elemental mapping confirmed the formation of a homogeneous Sn-based acidic ionic liquid with uniformly distributed constituent elements. The catalytic performance of this AIL was evaluated in the conjugate addition of aromatic and aliphatic amines to a broad range of α,β-unsaturated acceptors, including acrylates and acrylonitrile derivatives. Under optimized solvent-free conditions, using the ionic liquid itself as both catalyst and medium, the reactions proceeded smoothly at 60 °C, delivering the corresponding β-amino carbonyl and β-amino nitrile products, typically, in good to excellent isolated yields within short reaction times. Control experiments showed significantly reduced conversion in the absence of the ionic liquid, highlighting its essential catalytic role. Systematic optimization studies demonstrated that neat conditions outperformed reactions conducted in conventional solvents, such as water, ethyl acetate, methanol, acetonitrile, and toluene, underscoring the dual function of the AIL as an activating medium and catalyst. The method exhibits broad substrate scope and functional-group tolerance, accommodating electron-rich and electron-poor anilines, aliphatic amines, cyclic amines, and heterocyclic nucleophiles such as imidazole. Electron-donating substituents on aromatic amines generally enhanced reaction efficiency, while electron-withdrawing groups led to lower yields (Scheme 15).

A plausible reaction mechanism was proposed in which activation of the α,β-unsaturated acceptor occurs through coordination to the Lewis acidic Sn center and protonation by the acidic ionic liquid framework, thereby increasing electrophilicity at the β-carbon. Subsequent nucleophilic attack by the amine, followed by tautomerization, affords the observed β-amino products. This cooperative Brønsted–Lewis acid activation distinguishes the catalytic behavior of the Sn-based ionic liquid from purely Brønsted acidic or metal-free ionic liquid systems. From a green chemistry perspective, a key advantage of this protocol is the excellent recyclability of the acidic ionic liquid. The catalyst was readily separated from the reaction mixture and reused for at least five consecutive cycles with only a minor decrease in activity, demonstrating its operational stability and potential for sustainable applications. The combination of high efficiency, mild conditions, solvent-free operation, and catalyst reusability positions this Sn-based acidic ionic liquid as a valuable addition to the toolbox of ionic-liquid-mediated aza-Michael reactions.

### 3.2. Basic TSILs

Basic ionic liquids (BILs) have attracted considerable attention as efficient catalysts and reaction media for aza-Michael additions due to their dual functionality and tunable structures. In these systems, the basic ionic liquid not only replaces conventional organic solvents but also activates the nucleophile by enhancing the nucleophilicity of amines, thereby promoting C-N bond formation under mild conditions. Ionic liquids derived from 1,4-diazabicyclo[2.2.2]octane (DABCO) and tetramethylguanidine (TMG) have been successfully employed in aza-Michael reactions of both aliphatic and aromatic amines with α,β-unsaturated compounds. These catalysts often provide high yields, reduced reaction times, and simplified work-up procedures compared to traditional base catalysts. Moreover, their non-volatile nature and recyclability align well with green chemistry principles. Despite these advantages, many reported protocols require relatively high or stoichiometric amounts of basic ionic liquids, and in some cases show limited substrate scope or selectivity [31,33,34,35,57,58,59].

**Figure 1:**
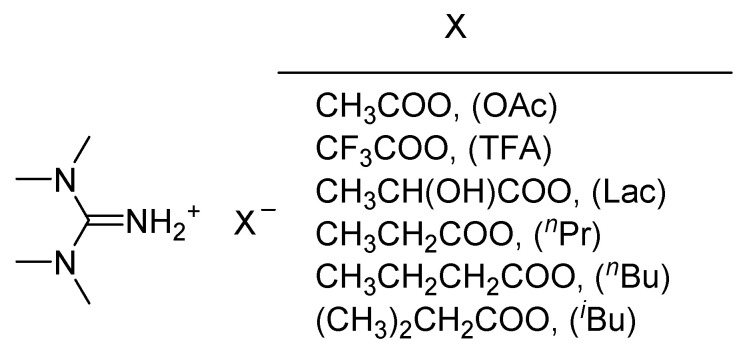
1,1,3,3-Tetramethylguanidine-based ionic liquids.

1,1,3,3-Tetramethylguanidine (TMG)-derived ionic liquids, prepared by simple neutralization reaction of TMG and acids (Figure 1), were evaluated as task-specific catalysts for aza-Michael additions [31]. The guanidine-based ionic liquids, exemplified by [TMG][Lac], can be classified as basic ionic liquids. Although formally protic ionic liquids, their catalytic behavior in aza-Michael reactions reflects the high intrinsic basicity of the guanidine framework combined with weakly coordinating carboxylate anions, enabling efficient base-promoted conjugate addition under solvent-free conditions.

**Scheme 16:**
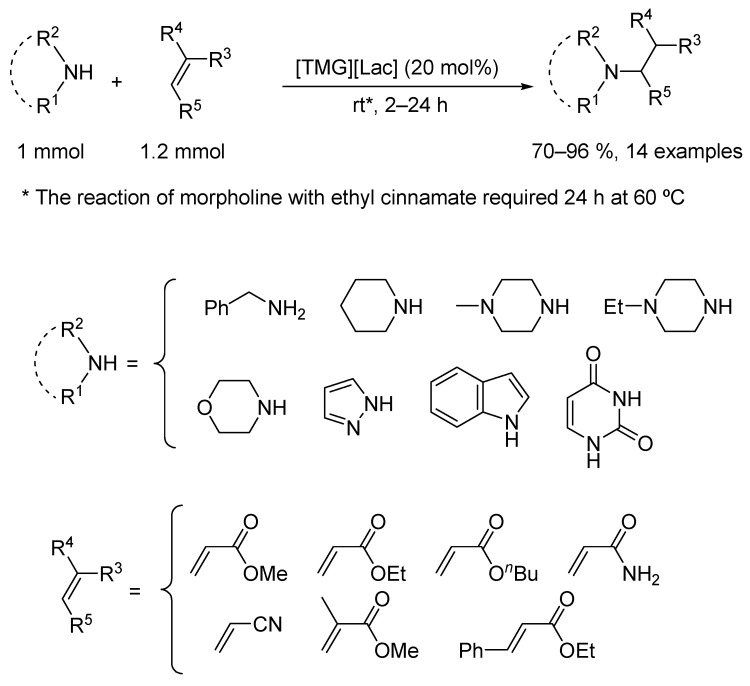
Aza-Michael addition using [TMG][Lac] as catalyst under solvent-free conditions.

The reaction of dibenzylamine with methyl acrylate was selected as a model system, and among several ionic liquids examined, [TMG][Lac] showed the highest activity in terms of yield and reaction rate. Solvent-free conditions were preferred for economic and environmental reasons, and an optimal catalyst loading of 0.2 equiv. was sufficient. In the absence of the ionic liquid, small conversion was observed, confirming its crucial catalytic role. Typically, reactions were carried out on 1.0 mmol scale of substrate with 1.2 equiv. of α,β-unsaturated compounds in the presence of 0.2 mmol of ionic liquid at room temperature, with yields of isolated product ranging from 70 to 96% in 2–24 h. The recyclability of [TMG][Lac] was confirmed through multiple reaction cycles without noticeable loss of activity, highlighting its potential as a green, efficient, and reusable catalyst for aza-Michael reactions (Scheme 16).

**Scheme 17:**
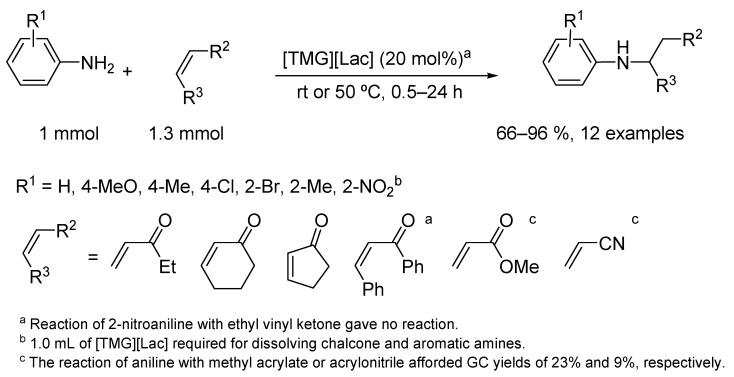
Aza-Michael addition of aromatic amines using [TMG][Lac] as catalyst.

To broaden the applicability of this methodology, various aromatic amines were evaluated as Michael donors under identical conditions with other α,β-unsaturated ketones, including ethyl vinyl ketone, 2-cyclohexen-1-one, 2-cyclopenten-1-one, and chalcone. Electron-donating substituents on the aromatic ring enhanced the Michael addition, whereas electron-withdrawing groups such as NO_2_ significantly reduced amine nucleophilicity. Notably, 2-cyclohexen-1-one and chalcone proved to be effective Michael acceptors, affording good to excellent isolated yields with a range of arylamines. In contrast, 2-cyclopenten-1-one exhibited lower reactivity. The addition of aniline to methyl acrylate or acrylonitrile was sluggish, giving only 23% and 9% yields, respectively, even after extending the reaction time to 24 h (Scheme 17).

**Scheme 18:**
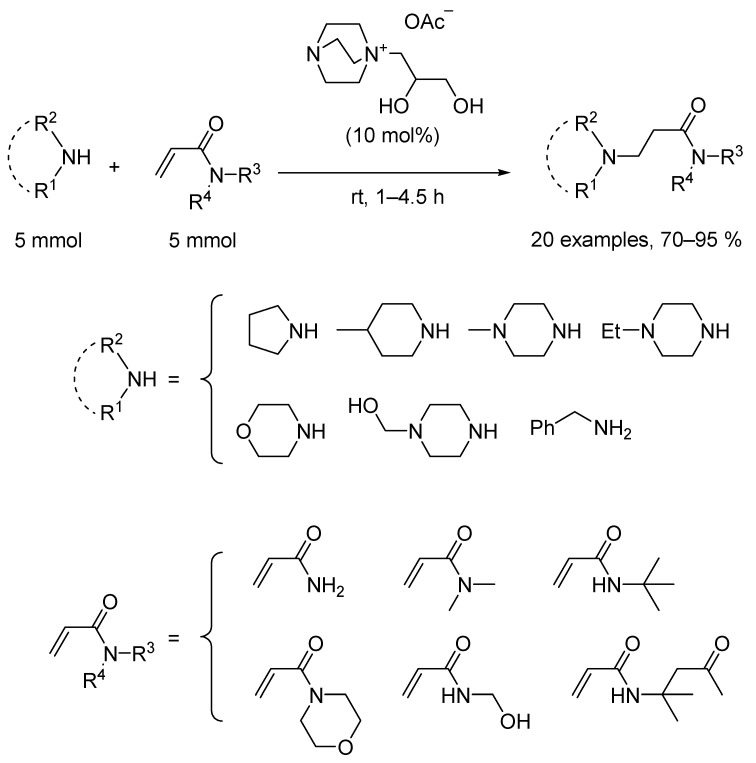
[DABCO-PDO][OAc] catalyzed aza-Michael addition of amines to α,β-unsaturated amides.

The same group later reported a family of task-specific, DABCO-based ionic liquids designed as efficient, recyclable organocatalysts for aza-Michael addition reactions under solvent-free conditions [33]. In their study, a series of ionic liquids derived from 1,4-diazabicyclo[2.2.2]octane (DABCO) and 3-chloro-1,2-propanediol were synthesized and evaluated for the conjugate addition of amines to α,β-unsaturated amides. Among the tested systems, [DABCO-PDO][OAc] emerged as the most effective catalyst, promoting reactions at room temperature with only 10 mol% loading and affording β-amino amide products in good to excellent yields within short reaction times. A broad substrate scope was demonstrated, including cyclic secondary amines (e.g., morpholine, piperidines, piperazines) and a wide range of *N*-substituted acrylamides. Steric and electronic effects of both the amine nucleophile and the Michael acceptor were systematically evaluated. Importantly, the ionic liquid could be recovered and reused up to eight times with minimal loss of activity, highlighting its robustness and practical applicability (Scheme 18).

Mechanistic studies combining FTIR spectroscopy and DFT calculations revealed a dual activation mode in which the basic DABCO moiety enhances amine nucleophilicity, while hydrogen-bonding interactions between the hydroxyl groups of the ionic liquid and the amide carbonyl activate the Michael acceptor.

**Scheme 19:**
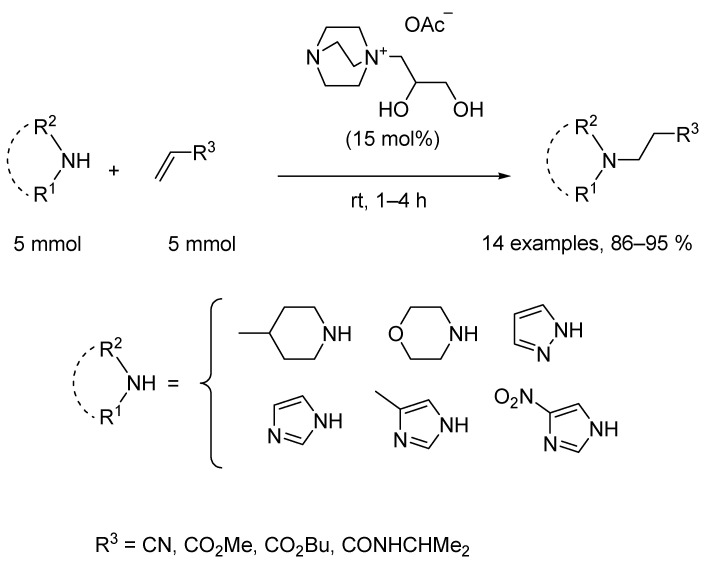
[DABCO-PDO][OAc] catalyzed aza-Michael addition of amines to α,β-unsaturated compounds.

In a subsequent study [34], the same research group extended this DABCO-based ionic liquid platform to the aza-Michael addition of secondary amines to a broader class of α,β-unsaturated compounds, including acrylates, acrylonitrile, and acrylamides. Again, [DABCO-PDO][OAc] was identified as the optimal catalyst, operating efficiently under solvent-free conditions at room temperature with 15 mol% catalyst loading. The reactions proceeded smoothly to give the corresponding β-amino esters and nitriles in high yields, often outperforming previously reported ionic liquid and non-ionic catalysts in terms of reaction time and efficiency (Scheme 19).

This study further substantiated the dual catalytic role of the DABCO-based ionic liquids through comparative experiments and computational analysis. The presence of hydroxyl functionalities in the cation was shown to be crucial for high activity, as simpler alkylated DABCO ionic liquids lacking hydrogen-bond donors exhibited inferior performance. Chemoselectivity studies also demonstrated preferential *N*- over *O*-addition in ambident nucleophiles, underscoring the controlled activation provided by the ionic liquid framework. The catalyst could be recycled at least six times without significant deactivation, reinforcing its suitability for sustainable synthesis.

Collectively, these contributions establish DABCO-based ionic liquids as a versatile and rationally designed class of organocatalysts for aza-Michael reactions. Their ability to combine basic activation, hydrogen-bonding interactions, solvent-free operation, and excellent recyclability makes them particularly attractive for green aza-Michael chemistry, especially in challenging systems such as α,β-unsaturated amides. These studies represent a clear example of how task-specific ionic liquids can be engineered to achieve cooperative catalysis beyond the capabilities of conventional molecular bases or simple ionic liquid media.

### 3.3. Heterogeneous Ionic Liquids: Supported and Polymeric Systems

Despite their name, polymeric ionic liquids are generally solid materials rather than liquids, as the term refers to their chemical lineage rather than their physical state. In these systems, ionic liquid motifs are covalently anchored within a polymer backbone or crosslinked network, which restricts ionic mobility and suppresses fluidity. In the context of aza-Michael reactions, this immobilization is particularly advantageous, since the catalytically active ionic sites responsible for activating electron-deficient alkenes or stabilizing charged intermediates are retained, while the catalyst operates in a heterogeneous mode. As a result, polymeric ionic liquids combine the reactivity and tunability characteristic of ionic liquids with the operational benefits of solid catalysts, including facile separation, recyclability, enhanced stability, and reduced leaching, making them especially attractive for sustainable aza-Michael methodologies.

**Figure 2:**
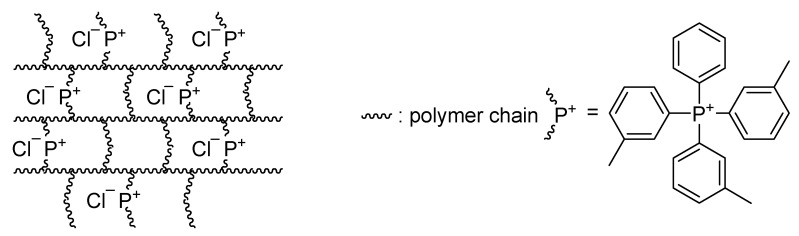
Schematic structure of the polymer.

Liang reported the development of a novel polymeric ionic liquid catalyst in which a benzyl(triphenyl)phosphonium ionic liquid moiety is inlaid directly into a hypercrosslinked polymer framework, rather than being grafted onto a preformed support [60]. The material was synthesized via quaternization of triphenylphosphine with *p*-xylylene dichloride, followed by SnCl_4_-catalyzed polycondensation with excess *p*-xylylene dichloride to generate a rigid, highly porous network. This synthetic strategy effectively avoids common drawbacks of supported ionic liquid phase catalysts, such as pore blocking, low ionic liquid loading, and leaching of the active species. The resulting polymer exhibited a high BET surface area (ca. 827 m^2^/g) together with a significant ionic liquid loading (ca. 1.40 mmol/g), ensuring good accessibility of the catalytically active phosphonium sites. Owing to the inlaid architecture, the ionic liquid moieties constitute part of the polymer backbone and are immobilized through multiple covalent linkages, which confers high structural stability and resistance to deactivation during catalysis (Figure 2).

**Scheme 20:**
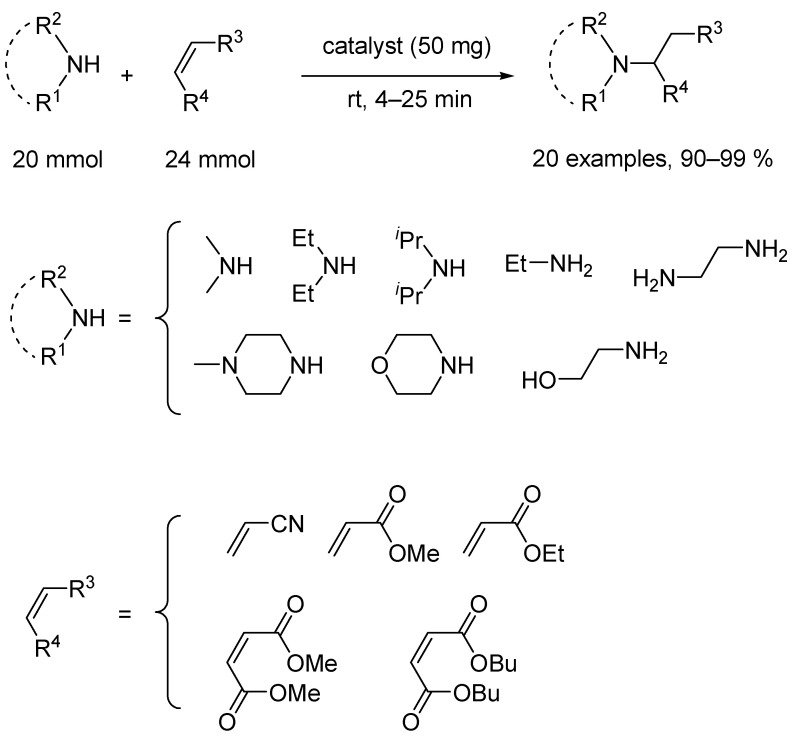
Aza-Michael reaction catalyzed by a crosslinked polymer with a benzyl(triphenyl)phosphonium IL moiety.

This polymeric ionic liquid showed excellent catalytic performance in aza-Michael addition reactions between a wide range of amines (primary, secondary, aliphatic, cyclic, and polyamines) and electron-deficient alkenes (acrylates, maleates, and acrylonitrile). Under mild conditions, reactions proceeded rapidly, typically within minutes, affording consistently high yields. Sterically hindered substrates reacted more slowly but still reached high conversions, highlighting the beneficial role of the high surface area and open pore structure in minimizing mass-transfer limitations (Scheme 20).

Importantly, the catalyst could be readily recovered by simple filtration and reused for at least seven cycles with negligible loss of activity or surface area, demonstrating clear advantages over homogeneous ionic liquids. Comparative studies showed that the polymeric ionic liquid outperformed its homogeneous phosphonium ionic liquid analogue, acidic ionic liquids, and conventional solid acids such as Amberlyst-15, underscoring the synergistic effect of ionic liquid functionality combined with a tailored porous polymer support This work illustrates an effective design principle for polymeric ionic liquids in aza-Michael reactions, where embedding the ionic liquid within a hypercrosslinked polymer matrix enables high activity, stability, and recyclability, thereby bridging the gap between homogeneous ionic liquid catalysis and practical heterogeneous systems.

In a closely related follow-up study, Li and co-workers extended this “IL-in-framework” design concept to a different cationic motif, reporting the synthesis of a hypercrosslinked porous polymer containing pyridinium ionic liquid structures derived from quaternized 4-vinylpyridine and p-xylylene dichloride. As in the 2015 work, the ionic liquid units were incorporated directly into the polymer backbone during SnCl_4_-catalyzed condensation, yielding a robust porous material with a high BET surface area (~667 m^2^ g^−1^) and an even higher IL loading (up to ~2.87 mmol g^−1^) [61]. The pyridinium-based polymer exhibited similarly excellent catalytic performance in aza-Michael reactions between a broad range of amines and activated alkenes. Under mild, solvent-free conditions at ambient temperature, high conversions (typically >95%) were achieved within minutes, including for sterically demanding amines, such as diethylamine and diisopropylamine. The catalyst showed excellent recyclability, maintaining activity over at least six consecutive cycles, with minimal loss of surface area or structural integrity. As in the earlier study, the heterogeneous polymer catalyst significantly outperformed the corresponding homogeneous ionic liquid and acidic IL benchmarks, underscoring the importance of framework-embedded IL architectures.

Taken together, the 2015 and 2016 papers establish a coherent design strategy for polymer-confined ionic liquid catalysts in aza-Michael chemistry. Both studies demonstrate that embedding ionic liquid moieties directly into a hypercrosslinked polymer framework, rather than simple surface grafting, maximizes IL loading while preserving high surface area and accessibility of active sites. These contributions collectively illustrate how rational materials design can overcome the classical drawbacks of homogeneous IL catalysis (recovery, viscosity, mass-transfer limitations) while retaining, or even enhancing, catalytic efficiency in aza-Michael reactions.

Ghasemi and co-workers reported an efficient aza-Michael-type addition protocol based on a supported ionic liquid phase (SILP) incorporating an anionic heteropoly acid, representing a further development in the immobilization of ionic-liquid catalysts for conjugate addition chemistry [62]. In this work, a phosphotungstate anion (PW) was incorporated into an imidazolium ionic liquid and subsequently immobilized on magnetic diatomaceous earth, affording a magnetically separable heterogeneous catalyst denoted as NPs-Fe_3_O_4_@DE@bmim_3_PW. The catalyst combines the high activity of heteropoly-acid-based ionic liquids with the operational advantages of heterogeneous and magnetically recoverable systems.

**Scheme 21:**
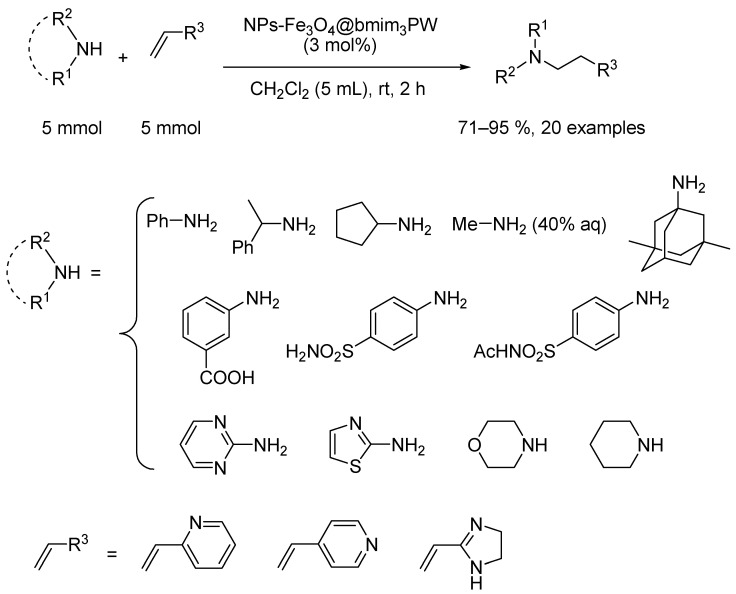
NPs-Fe_3_O_4_@DE@bmim_3_PW catalyzed aza-Michael-type addition of various amines to vinyl compounds.

Systematic catalyst screening demonstrated that heteropoly acids outperform conventional Lewis acids and simple Brønsted acids in aza-Michael-type additions, owing to their dual Brønsted–Lewis acidic character. Among the tested systems, the supported ionic liquid incorporating phosphotungstate exhibited the highest activity in the conjugate addition of amines to nitrogen-containing vinyl substrates such as 2-vinylpyridine, 4-vinylpyridine, and 2-vinylimidazole. Under mild conditions (room temperature, 3 mol% catalyst), a wide range of amines, including primary alkylamines, secondary amines, and deactivated anilines, were converted to the corresponding β-amino products in good to excellent isolated yields, typically within 2 h (Scheme 21).

A notable advantage of this catalytic system is its excellent recyclability and ease of separation. The magnetic SILP catalyst was readily removed from the reaction mixture using an external magnetic field and reused for at least five consecutive cycles without significant loss of activity. Control experiments confirmed that the supported IL catalyst outperformed both the unsupported ionic liquid and the magnetic support alone, indicating a synergistic effect between the ionic liquid phase, the heteropoly-acid anion, and the solid support. Moreover, immobilization of the acidic ionic liquid effectively suppressed undesirable side reactions such as vinyl polymerization, which are often observed with conventional Brønsted acids.

Collectively, these three studies illustrate the versatility of supported and polymeric ionic-liquid platforms as a powerful approach for achieving high efficiency, operational simplicity, and sustainability in aza-Michael chemistry.

### 3.4. Miscellaneous TSILs

While early applications described DBU-based ionic liquids as basic ILs, mechanistic studies later demonstrated that their superior performance in aza-Michael reactions originates from bifunctional, protic acid-base catalysis rather than from pure base strength [63].

**Scheme 22:**
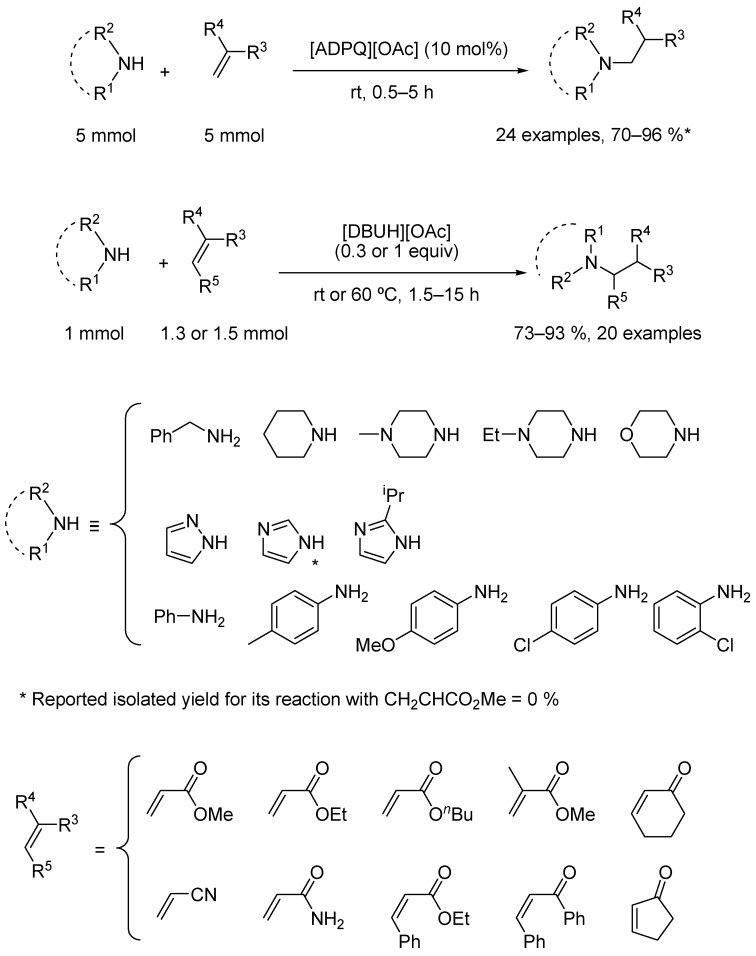
Aza-Michael reaction in [DBUH][OAc] TSIL.

Ying and co-workers reported a first study of aza-Michael reactions promoted by a DBU-derived ionic liquid, 1,8-diazabicyclo[5.4.0]undec-7-en-8-ium acetate ([DBUH][OAc]), used as a recyclable catalyst under solvent-free conditions at room temperature [59]. This task-specific ionic liquid efficiently catalyzed the conjugate addition of a broad range of aliphatic and aromatic amines to α,β-unsaturated esters, nitriles, amides, enones, and chalcones, affording the corresponding β-amino products in good to excellent yields. Notably, [DBUH][OAc] enabled aza-Michael reactions of aromatic amines under mild conditions and consistently outperformed both free DBU and imidazolium-based hydroxide ionic liquids. The catalyst could be readily recovered and reused for at least six cycles without loss of activity, and the methodology was demonstrated on a 100 mmol scale, highlighting its practical and sustainable character (Scheme 22).

**Figure 3:**
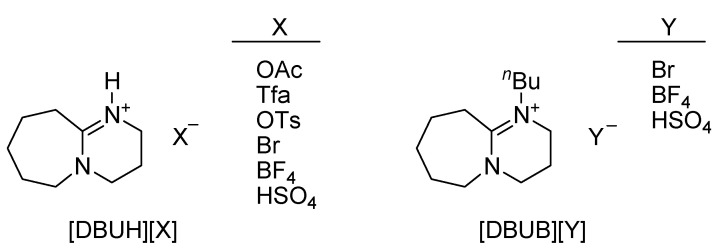
DBU-based ionic liquids.

Although [DBUH][OAc] was initially regarded as a basic ionic liquid, it is more accurately classified as a DBU-based protic ionic liquid (Figure 3) with weak to moderate intrinsic basicity. The conjugate base (OAc^-^) and the DBU/DBUH^+^ equilibrium can still promote basic catalytic activity. However, its high catalytic efficiency cannot be attributed solely to Brønsted basicity but is instead associated with ionic-liquid-specific effects, including enhanced amine nucleophilicity, hydrogen-bonding interactions, and cooperative activation of the Michael acceptor, as later confirmed by detailed mechanistic studies on DBU-based ionic liquids [63].

**Scheme 23:**
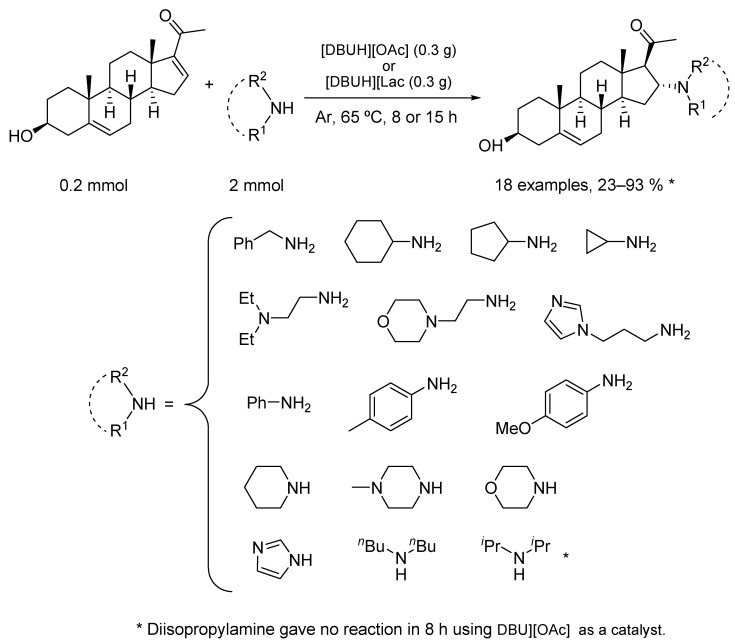
Aza-Michael reaction of 16-dehydropregnenolone in DBU-based ILs.

Subsequently, Szánti-Pintér and co-workers reported the use of [DBUH][OAc] and [DBUH][Lac] as efficient catalysts and reaction media for aza-Michael additions to steroidal Michael acceptors [35]. Although these systems are formally classified as protic ionic liquids, they exhibit pronounced basic catalytic behavior, which can be attributed to the strong basic character of the DBU-derived framework in combination with the weakly acidic nature of the counterions. In this study, the conjugate addition of a wide range of primary and secondary amines to 16-dehydropregnenolone was performed under solvent-free conditions using the ionic liquid as both solvent and base catalyst. The reactions proceeded smoothly at moderate temperature, affording 16α-aminopregnenolone derivatives in moderate to good isolated yields. Importantly, the ionic liquid catalyst could be recovered and reused for at least five cycles without significant loss of activity, highlighting its operational stability and suitability for sustainable steroid functionalization (Scheme 23).

This work demonstrated that DBU-based ionic liquids are particularly effective for challenging aza-Michael reactions involving sterically hindered, electron-deficient steroidal enones. Both aliphatic and aromatic amines, including heterocyclic nucleophiles, were successfully employed. Comparison with reactions conducted in the absence of ionic liquid confirmed the essential catalytic role of the DBU-based IL in enhancing amine nucleophilicity and activating the conjugated enone system.

In a complementary mechanistic study, Cândido and co-workers systematically investigated the role of DBU-based protic, bifunctional (acid-base) ionic liquids, and in some cases even mildly acidic PILs (Figure 3), ionic liquids in aza-Michael reactions using a combination of experimental catalysis, electrospray ionization mass spectrometry (ESI-MS), and density functional theory (DFT) calculations [63].

**Scheme 24:**
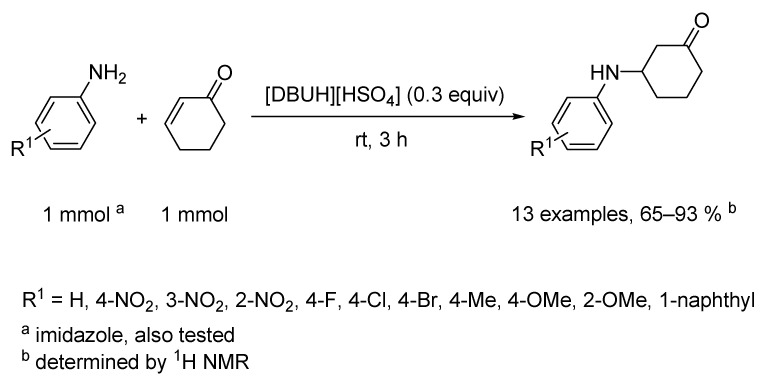
Aza-Michael addition catalyzed by [DBUH][HSO_4_] TSIL.

Various protic and aprotic DBU-based ILs were evaluated in the conjugate addition of aromatic amines to cyclohex-2-en-1-one under solvent-free, room-temperature conditions. Protic DBU-based ILs such as [DBUH][OAc], [DBUH][TFA], and particularly [DBUH][HSO_4_] exhibited excellent catalytic activity, affording high conversions even with poorly nucleophilic aromatic amines bearing strong electron-withdrawing substituents (Scheme 24).

Mechanistic investigations revealed that efficient catalysis requires a cooperative interaction between the protic DBUH^+^ cation and the anion. ESI-MS “ion-fishing” experiments and DFT calculations showed that hydrogen bonding between the anion and the amine nucleophile, combined with activation of the Michael acceptor by the acidic proton of the cation, lowers the activation barrier for C-N bond formation. Aprotic DBU-based ILs lacking an acidic proton were markedly less effective, underscoring the importance of proton-assisted activation in these systems. Based on these insights, the authors proposed a general catalytic model that rationalizes the superior performance of protic DBU-based ILs and provides guidelines for the rational design of next-generation ionic liquid organocatalysts.

**Figure 4:**
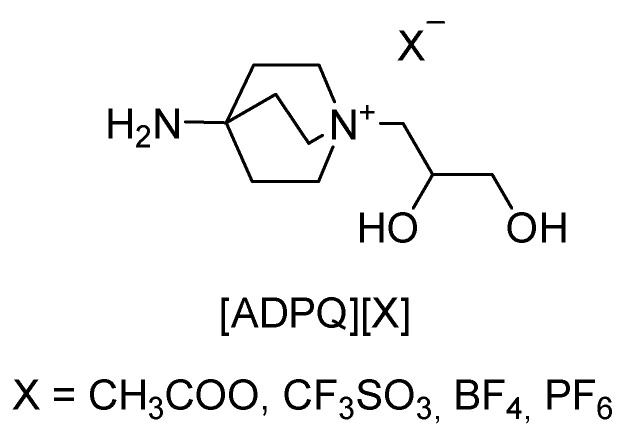
Aminoquinuclidine-based ILs.

Gao and Gao reported the development of a novel class of task-specific ionic liquids based on a 4-aminoquinuclidine scaffold, exemplified by [ADPQ][OAc], and their application as efficient and recyclable catalysts for aza-Michael addition reactions [64]. These ionic liquids were synthesized through quaternization of 4-aminoquinuclidine followed by anion exchange to introduce acetate, yielding hydroxyl-functionalized ammonium salts that operate effectively under solvent-free conditions at room temperature (Figure 4).

**Scheme 25:**
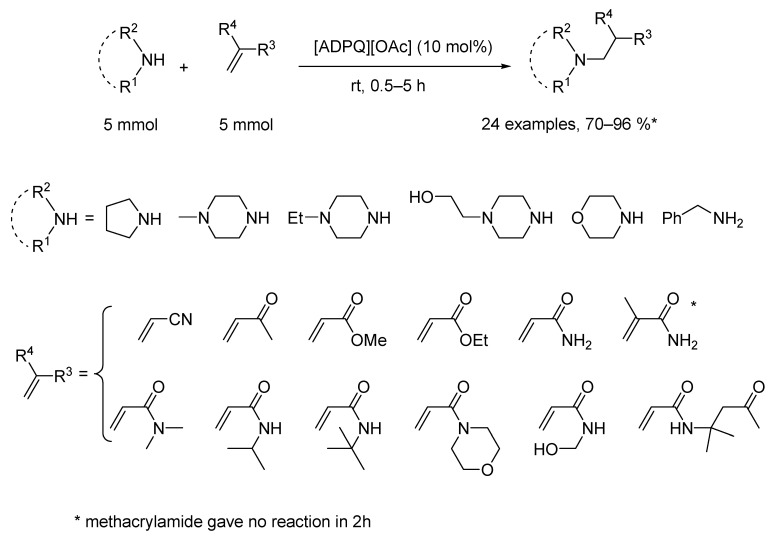
[ADPQ][OAc] catalyzed aza-Michael addition.

The catalytic performance of [ADPQ][OAc] was evaluated using the conjugate addition of a wide range of amines to α,β-unsaturated amides, esters, nitriles, and enones. Secondary cyclic amines such as morpholine, piperazines, pyrrolidine, and substituted piperidines reacted smoothly with acrylamide and related Michael acceptors, typically affording β-amino products in good to excellent isolated yields within short reaction times (often 0.5–3 h). Primary amines, including benzylamine, were also compatible, although slightly lower yields were observed in some cases. Steric effects at either the amine or the Michael acceptor were shown to influence reaction rates and conversions, particularly for substituted acrylamides. Optimization studies identified 10 mol% catalyst loading as optimal, and the reactions proceeded efficiently without the need for additional solvents (Scheme 25).

The ionic liquid could be readily recovered after product isolation and reused for at least six consecutive cycles without a noticeable decrease in catalytic activity. The methodology was also demonstrated on a 100 mmol scale, highlighting its potential practical applicability and operational robustness.

Mechanistic investigations indicated that the catalytic activity of [ADPQ][OAc] does not arise from Brønsted basicity or acidity. Instead, the authors proposed a hydrogen-bond-driven activation mechanism in which the hydroxyl groups on the ionic-liquid cation interact with the carbonyl group of the α,β-unsaturated substrate, increasing its electrophilicity toward nucleophilic amine addition. This interpretation was supported by ^13^C NMR studies showing significant chemical-shift changes upon mixing the ionic liquid with methyl acrylate, as well as by comparative experiments demonstrating higher activity for ILs bearing multiple hydroxyl groups.

Overall, this work establishes 4-aminoquinuclidine-based ionic liquids as a distinct class of hydrogen-bond-functionalized task-specific ionic liquids for aza-Michael reactions. Their high efficiency under mild, solvent-free conditions, broad substrate scope, and excellent recyclability make them a valuable complement to acidic, basic, and protic ionic-liquid systems commonly employed in aza-Michael chemistry.

## 4. Bio-Inspired and Bio-Sourced Ionic Liquids

Morimoto and co-workers reported the use of imidazolium-based ionic amino acids, [emim][AA], as organocatalysts for the aza-Michael addition of aromatic amines to α,β-unsaturated ketones, representing one of the earliest examples of amino acid-derived ionic liquids applied to this transformation [65]. In these systems, the anion originates from naturally occurring amino acids (e.g., glycine, proline, phenylalanine), while the ethylmethylimidazolium cation remains fully synthetic, resulting in partially bio-inspired but not fully bio-based ionic liquids. Among the catalysts examined, [emim][Gly], [emim][Br], [emim][OH], and Gly, the amino acid-based IL proved most effective, and was chosen as the model reaction to determine the scope of this system. Control experiments demonstrated that both ions were essential and neither the amino acid alone nor imidazolium salts lacking the amino acid anion promoted the reaction, highlighting the cooperative role of the ionic liquid architecture.

**Scheme 26:**
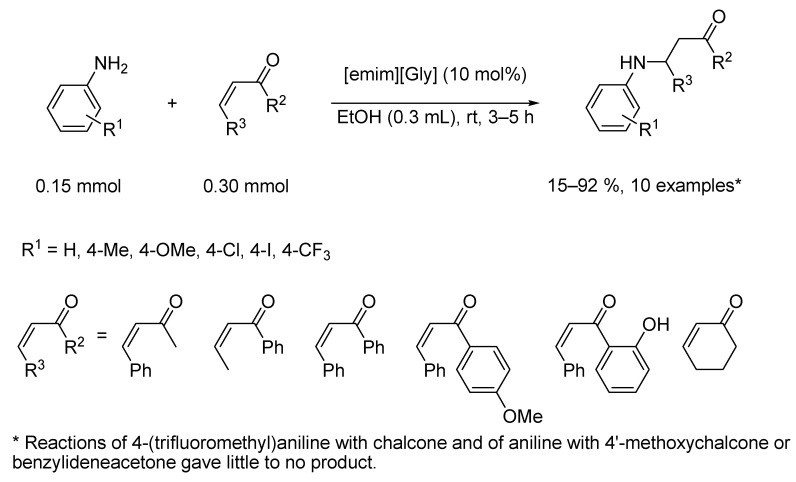
Aza-Michael reactions catalyzed by [emim][Gly].

The catalytic system exhibited moderate substrate scope, favoring electron-rich anilines and simple chalcone derivatives, while electron-poor amines and cyclic enones showed reduced reactivity. Notably, the ionic amino acid catalysts could be recovered and reused at least three times without loss of activity (Scheme 26).

Subsequently, a series of [emim][AA] catalysts from 20 natural amino acids were evaluated as catalysts in the aza-Michael reaction and the enantiomeric excess was determined by chiral HPLC for chiral aa-based ILs. The [emim][L-Pro] catalyst gave the desired product in the highest yield, whereas [emim][L-Phe] provided the highest enantiomeric excess (90%) with a 45% yield. Mechanistic investigations supported a non-covalent activation pathway, involving hydrogen-bond-assisted organization of the nucleophile rather than classical iminium ion catalysis, as evidenced by ESI-MS studies.

From a green chemistry perspective, this work constitutes an important transitional step toward sustainable ionic liquids for aza-Michael reactions. The use of renewable amino acid anions improves biodegradability and reduces toxicity relative to conventional ILs, yet the presence of a synthetic imidazolium cation prevents classification as a fully bio-based system.

Kumar and co-workers reported the use of choline hydroxide ([Ch]OH) as a green, basic ionic liquid catalyst for the aza-Michael addition of nitrogen nucleophiles to activated α,β-unsaturated compounds under mild conditions [58]. In this study, commercially available aqueous choline hydroxide was employed both as catalyst and reaction medium, enabling efficient C–N bond formation at room temperature without the need for volatile organic solvents. Reaction optimization using the model coupling of imidazole with ethyl acrylate demonstrated that 1.0 mmol of [Ch]OH was sufficient to achieve near-quantitative conversion, affording the desired aza-Michael adduct in up to 96% isolated yield within 40 min in water. Lower catalyst loadings resulted in diminished yields, highlighting the importance of adequate hydroxide availability for effective activation of the nucleophile.

**Scheme 27:**
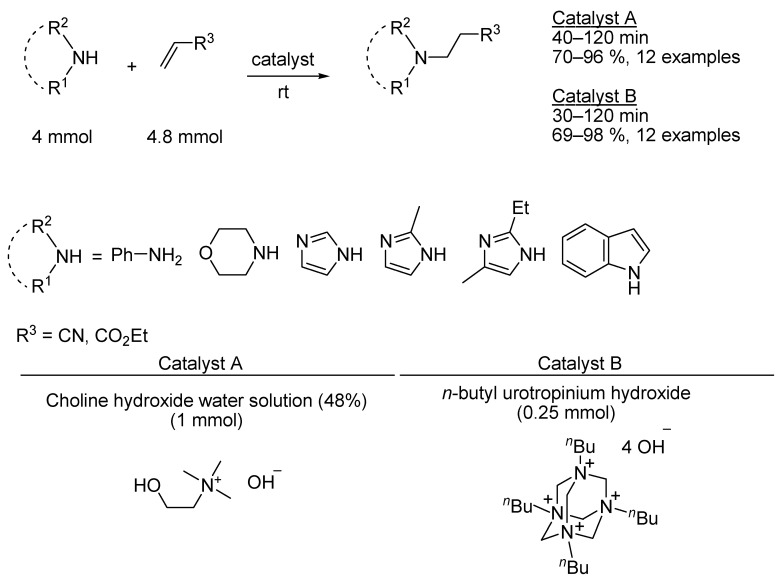
Aza-Michael reaction with cholinium hydroxide as solvent and catalyst.

Under the optimized conditions, the scope of the [Ch]OH-catalyzed protocol was evaluated with a range of *N*-nucleophiles, including imidazole, substituted imidazoles, indole, morpholine, and aniline, in combination with ethyl acrylate and acrylonitrile as Michael acceptors. Imidazole and its derivatives consistently gave the highest yields (typically >90%), reflecting their enhanced nucleophilicity in the strongly basic ionic liquid environment. Less nucleophilic amines such as aniline reacted more slowly and afforded comparatively lower yields, though still under mild and operationally simple conditions (Scheme 27).

A key advantage of choline hydroxide highlighted in this work is its biocompatibility, low cost, and biodegradability, distinguishing it from many conventional basic ionic liquids. Importantly, the aqueous [Ch]OH phase could be recovered and reused for at least five consecutive cycles without significant loss of catalytic activity, satisfying key criteria of sustainability and process economy. The authors attribute the high efficiency of [Ch]OH to the strong basicity of the hydroxide anion combined with the favorable physicochemical properties of the choline cation, which facilitate nucleophile activation and stabilization of reaction intermediates in aqueous media.

**Figure 5:**
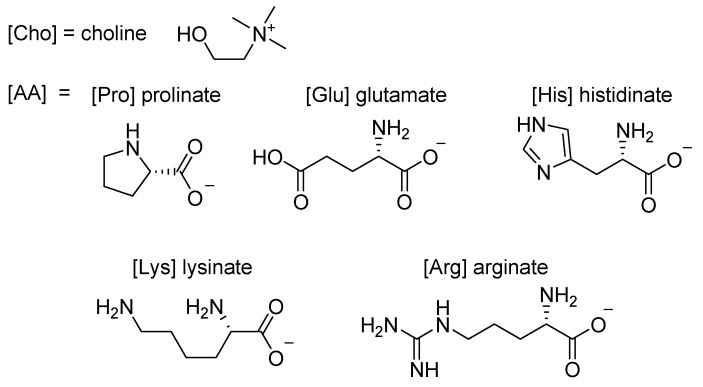
Choline-amino acid ILs studied [Cho][AA].

In a 2024 paper, Izquierdo et al. reported that aza-Michael reactions can be catalyzed by cholinium α-amino carboxylates, in which both the cation (choline) and the anion (amino acid) are bio-derived, thus achieving a genuinely fully bio-based ionic liquid platform. Cholinium amino-acid ILs ([Cho][AA]) are prepared from choline and natural amino acids and display low toxicity and biodegradability. Izquierdo et al. demonstrated that cholinium α-amino carboxylates efficiently catalyze aza-Michael additions of amines to acrylates at low loadings (typically 5–10 mol%) with excellent yields and significant rate enhancement compared to conventional ILs or neat conditions [66]. Choline was paired with arginine, lysine, histidine, proline, and glutamic acid (Figure 5).

**Scheme 28:**
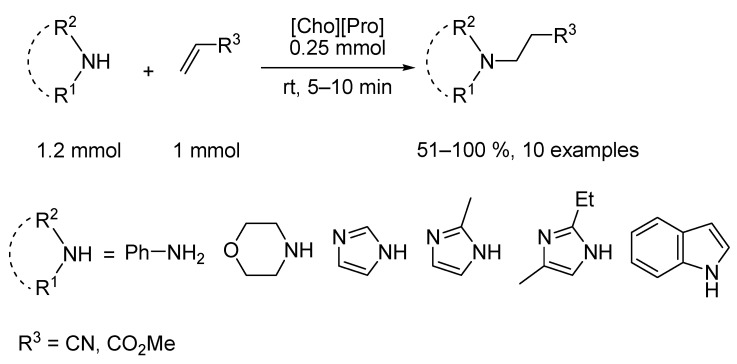
Aza-Michael reaction catalyzed by [Cho][Pro].

In particular, [Cho][Pro] stands out, likely because of cooperative hydrogen bonding and conformational restriction, as supported by DFT analyses of the transition state (Scheme 28).

These ChAAILs often operate under solvent-free conditions and can be reused several times, with minimal loss of activity. Their high structural organization and strong yet labile hydrogen-bond networks seem critical to their performance. In another study, the authors compared the performance of [Cho][Pro] with that of hydrothermal carbons prepared from biomass waste, montmorillonite K10, and K10+[Cho][Pro] in model aza-Michael reactions. [Cho][Pro] alone or in combination with K10, still stood as the most efficient catalytic system, achieving complete conversion in a few minutes [67].

Lu and Brook extended this concept to polymer modification, using ChAAILs as both reagents and self-solvating media to attach amino acids to acrylate-functionalized polymers via aza-Michael reactions [68]. Their work demonstrates protecting-group-free incorporation of amino acids into hydrophilic polyethers and hydrophobic silicones, enabling the design of biocompatible, degradable materials. Importantly, ChAAILs circumvent solubility problems between zwitterionic amino acids and hydrophobic polymers and avoid external solvents.

## 5. Conclusions and Outlook

The body of work reviewed herein demonstrates that ionic liquids have evolved from whimsical reaction media into highly versatile tools for the promotion and catalysis of aza-Michael reactions. Early studies established that conventional imidazolium-based ionic liquids can act as non-innocent solvents, stabilizing polar intermediates, enhancing electrophilicity, and enabling aza-Michael additions under milder conditions than those required in molecular solvents. Subsequent developments have shown that the true potential of ionic liquids lies in their structural tunability, which has enabled the rational design of task-specific systems capable of providing Brønsted acidity, basicity, hydrogen-bonding ability, or cooperative acid-base activation within a single ionic framework.

A key outcome of recent research is the recognition that many ionic liquids operate through multifaceted and cooperative mechanisms rather than simple solvent or acid–base effects. In particular, guanidine-, DABCO-, and DBU-derived ionic liquids illustrate how these compounds, when incorporated into ionic environments, can promote aza-Michael reactions through bifunctional activation pathways that enhance both nucleophilicity and electrophilicity. Likewise, hydrogen-bond-functionalized and supported or polymer-embedded ionic liquids demonstrate that effective catalysis can be achieved without relying on strong Brønsted acidity or basicity, while simultaneously addressing practical issues related to catalyst recovery and recyclability.

From a sustainability perspective, the reviewed studies reveal both opportunities and limitations. While ionic liquids offer clear advantages in terms of reduced volatility, catalyst recyclability, and process integration, their environmental impact cannot be assumed a priori and must be evaluated in the context of synthesis, toxicity, and end-of-life considerations. Recent advances, such as bio-derived ionic liquids, CO_2_-switchable systems, and heterogenized ionic-liquid catalysts, represent promising strategies to align aza-Michael chemistry more closely with green chemistry principles.

Looking forward, several challenges and opportunities remain. Greater emphasis on mechanistic understanding, supported by spectroscopic, kinetic, and computational studies, will be essential to guide the rational design of next-generation ionic liquids. Comparative studies benchmarking ionic-liquid-based systems against conventional organocatalysts under identical conditions would further clarify their practical advantages. Finally, the integration of ionic liquids into continuous-flow processes, recyclable catalytic platforms, and circular reaction schemes is expected to play an increasingly important role in translating aza-Michael methodologies from laboratory-scale demonstrations to sustainable synthetic applications.

Overall, ionic liquids have established themselves as powerful and adaptable components of aza-Michael chemistry, and continued innovation in ionic-liquid design and process engineering is likely to further expand their impact on efficient and sustainable C-N bond formation.

## Data Availability

Not applicable.

## Abbreviations

AIL	acidic ionic liquid
Arg	arginine/arginate
BET	Brunauer-Emmett-Teller
BIL	basic ionic liquid
bmim	1-butyl-3-methylimidazolium
bpy	1-butylpyridinium
ChAAIL	cholinium amino acid ionic liquid
Cho	choline
DABCO	1,4-diazabicyclo[2.2.2]octane
DABCO-PDO	1-(propane-1,2-diol-3-yl)-1,4-diazabicyclo[2.2.2]octan-1-ium
damim	1,3-diallyl-2-methylimidazolium
dbmim	1,3-di-*n*-butyl-2-methylimidazolium
DBU	1,8-diazabicyclo[5.4.0]undec-7-ene
DBUB	8-*n*-butyl-1,8-diazabicyclo[5.4.0]undec-7-en-8-ium
DBUH	1,8-diazabicyclo[5.4.0]undec-7-en-8-ium
DDPSA	3-(dimethyldodecylammonium)propane-1-sulfonic acid
DFT	density functional theory
FT-IR	Fourier-transform infrared spectroscopy
GC	gas chromatography
GC-MS	gas chromatography–mass spectrometry
Glu	glutamic acid/glutamate
His	histidine/histidinate
Hmim	1-methylimidazolium
IL	ionic liquid
Lac	lactic acid/lactate
Lys	lysine/lysinate
NMR	nuclear magnetic resonance
OAc	acetate
PIL	protic ionic liquid
Pro	proline/prolinate
RTIL	room-temperature ionic liquid
SILP	supported ionic liquid phase
TBPSA	3-(tri-*n*-butylammonium)propane-1-sulfonic acid
TEBSA	4-(triethylammonium)butane-1-sulfonic acid
TEPSA	3-(triethylammonium)propane-1-sulfonic acid
TFA	trifluoroacetic acid/trifluoroacetate
TMBSA	4-(trimethylammonium)butane-1-sulfonic acid
TMG	tetramethylguanidinium
TMPSA	3-(trimethylammonium)propane-1-sulfonic acid
TSIL	task-specific ionic liquid
XRD	X-ray diffraction
